# Pathogenesis of Tuberculosis: Interplay Between Host Antituberculosis Immunity and Immune Evasion Strategies of *Mycobacterium Tuberculosis*

**DOI:** 10.1002/mco2.70884

**Published:** 2026-07-25

**Authors:** Xiaojuan Zhang, Ying Liu, Yilin Hou, Hongxia Niu

**Affiliations:** ^1^ Key Laboratory of Blood‐stasis‐toxin Syndrome of Zhejiang Province School of Basic Medical Science Zhejiang Chinese Medical University Hangzhou Zhejiang China

**Keywords:** host‐directed therapy, immunity, immune evasion, pathogenesis, tuberculosis

## Abstract

Tuberculosis remains one of the leading causes of death from a single infectious pathogen worldwide, despite the availability of antituberculosis chemotherapy and Bacillus Calmette‐Guérin vaccination. Tuberculosis pathogenesis is driven by a dynamic and prolonged interplay between host protective immunity and the immune evasion strategies of Mycobacterium tuberculosis (*M. tuberculosis*). During infection, bidirectional host–*M. tuberculosis* interactions, together with immunometabolic and epigenetic reprogramming, shape granuloma formation and organization, fibrotic encapsulation, liquefactive necrosis, and ultimately determine disease progression and transmission. However, a systematic framework linking host protective immunity, *M. tuberculosis* immune evasion, and granuloma evolution across distinct stages of infection remains incomplete. In this review, we summarize the host–*M. tuberculosis* interactions that shape early innate immune recruitment, adaptive immune activation, granuloma formation and remodeling, persistent infection, granuloma breakdown, and progression to active tuberculosis. We further integrate emerging concepts of metabolic reprogramming, epigenetic reprogramming, trained immunity, tissue‐resident immunity, and myeloid‐derived immunosuppressive networks. Finally, we discuss how these mechanistic insights may inform the design of next‐generation tuberculosis vaccines and adjunctive therapies. This review establishes a comprehensive conceptual framework for understanding tuberculosis immunopathogenesis and provides guidance for the development of antituberculosis vaccines and therapeutic strategies.

## Graphical abstract

***Mycobacterium tuberculosis*–host interactions and infection outcomes**. Following initial infection, alveolar macrophages phagocytose the pathogen, prompting the recruitment of innate immune cells including neutrophils, NK cells, and interstitial macrophages. Subsequently, dendritic cells migrate to the lymph nodes for antigen presentation, driving the activation of naïve T cells and culminating in the formation of granulomas to contain the infection. To counteract host defenses, *M. tuberculosis* deploys multifaceted evasion strategies. It induces phagosome arrest via factors such as ManLAM, SapM, and PtpA, and resists oxidative killing by suppressing NOX2‐mediated ROS/RNS production, thereby facilitating intracellular survival. Furthermore, the pathogen actively manipulates host cell death (inhibiting apoptosis while promoting necrosis), impairs dendritic cell maturation and antigen presentation, and drives immune exhaustion characterized by the upregulation of coinhibitory receptors (PD‐1, TIM‐3, and LAG‐3).The dynamic and prolonged interplay between host immunity and *M. tuberculosis* evasion dictates the ultimate trajectory of the disease. Robust immune containment leads to bacterial clearance or latent infection within stable granulomas. Conversely, driven by altered host status, coinfection, or physiological stress, this balance can undergo a dynamic transition, resulting in granuloma disruption, liquefactive necrosis, and progression to active tuberculosis.

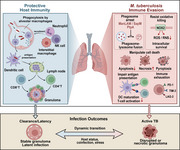

## 1 Introduction

Tuberculosis is one of the oldest recorded infectious diseases in human history; nevertheless, it remains a major global public health challenge that continues to pose a serious threat to human health [1]. According to the latest World Health Organization (WHO) report, there were an estimated 10.7 million new tuberculosis cases worldwide in 2024, and tuberculosis‐related deaths have surpassed those associated with acquired immunodeficiency syndrome, once again making tuberculosis the deadliest infectious disease caused by a single pathogen [2]. Notably, in high‐burden regions such as sub‐Saharan Africa and Southeast Asia, the high prevalence of human immunodeficiency virus (HIV) infection, together with the ongoing spread of drug‐resistant *Mycobacterium tuberculosis* (*M. tuberculosis*) strains, act synergistically to further increase tuberculosis incidence and mortality [3].

In 1944, streptomycin was first introduced for the treatment of active pulmonary tuberculosis, achieving effective bactericidal activity against *M. tuberculosis* and breaking the long‐standing notion that tuberculosis was an incurable disease [4]. This milestone marked the beginning of the era of antituberculosis chemotherapy. However, subsequent clinical studies revealed that streptomycin exhibits limited efficacy against nonreplicating or dormant bacilli and is prone to the rapid emergence of drug resistance [5]. Consequently, combination chemotherapy regimens were gradually established, incorporating additional agents such as isoniazid, rifampicin, and pyrazinamide, which together formed the foundation of modern tuberculosis treatment [6]. Despite these advances, the clinical management of tuberculosis continues to face major challenges due to the escalating burden of drug‐resistant disease. Drug resistance has progressed from mono‐resistant tuberculosis to multidrug‐resistant tuberculosis (MDR‐TB), and further to extensively drug‐resistant tuberculosis (XDR‐TB). It was reported that in 2023, only approximately 44% of the estimated 400,000 MDR‐TB cases were diagnosed and treated, with an overall treatment success rate of 68% [2]. XDR‐TB is associated with even poorer treatment outcomes, with a global cure rate of only 44.2% [7].

In addition to chemotherapy, vaccination has also played a critical role in tuberculosis control. In 1921, the Bacillus Calmette‐Guérin (BCG) vaccine was first administered to humans, marking the beginning of its clinical use [8]. Since then, BCG has become the cornerstone of tuberculosis prevention worldwide and remains the only WHO‐recommended tuberculosis vaccine, particularly for neonatal immunization in high‐burden regions [9]. Although BCG confers substantial protection against severe forms of childhood tuberculosis, especially tuberculous meningitis and miliary tuberculosis, its protective efficacy against adult pulmonary tuberculosis is limited and variable [10]. Collectively, the limitations in both current chemotherapy and vaccination strategies underscore the urgent need to develop more effective therapeutic and preventive approaches to achieve sustained control and eventual elimination of tuberculosis.

A comprehensive understanding of the complex interactions between *M. tuberculosis* and the host immune system is essential for identifying novel therapeutic targets and has important implications for tuberculosis prevention. Accumulating evidence indicates that, during the early stage of infection, *M. tuberculosis* is recognized and phagocytosed by alveolar macrophages (AMs) [11, 12]. Subsequently, natural killer (NK) cells secrete interferon‐γ (IFN‐γ) to enhance macrophage bactericidal activity; γδ T cells recognize mycobacterial lipid antigens and produce cytokines; and neutrophils capture bacilli through the formation of neutrophil extracellular traps (NETs) [13]. Concurrently, dendritic cells (DCs) present mycobacterial antigens to CD4^+^ T cells, inducing a Th1‐polarized immune response in which IFN‐γ and tumor necrosis factor‐α (TNF‐α) drive *M. tuberculosis*‐specific antimicrobial activity [14]. CD8^+^ T cells further contribute by directly lysing infected host cells via perforin‐ and granzyme‐mediated cytotoxicity, thereby limiting intracellular bacterial replication [15]. However, under sustained immune pressure, *M. tuberculosis* has evolved multiple immune evasion strategies that enable long‐term persistence within the host [16, 17]. Disruption of the delicate balance between host immunity and bacterial countermeasures represents a central determinant of tuberculosis pathogenesis. Excessive immune evasion by *M. tuberculosis* or insufficient host immune responses permits uncontrolled bacterial expansion, ultimately leading to disease progression. Thus, *M. tuberculosis* infection is characterized by a highly complex and dynamic interaction with the host immune system, which not only determines whether infection is cleared or established but also critically shapes disease progression and clinical outcomes.

In recent years, rapid advances in tuberculosis immunology have profoundly accelerated our understanding of the interactions between *M. tuberculosis* and the host immune system. In this review, we focus on the dynamic immunological tug‐of‐war between host antituberculosis immune responses and *M. tuberculosis* immune evasion strategies during the formation and progression of tuberculosis granulomas, and systematically synthesize the immunological mechanisms underlying tuberculosis onset and progression. We first provide an overview of the key components of host anti‐*M. tuberculosis* immunity, encompassing innate immune recognition, activation of adaptive immune responses, and the establishment of immunoregulatory networks. We then highlight the major immune evasion strategies employed by *M. tuberculosis* at the cellular, molecular, and tissue levels. Building on this framework, we further discuss how the sustained interplay between immune activation and immunosuppression drives granuloma evolution, maintenance of latent infection, immune exhaustion, and disease progression. Finally, integrating the most recent advances in the field, we explore the implications of this bidirectional interaction framework for tuberculosis vaccine development and adjunctive therapies, thereby providing a systematic conceptual roadmap for future research.

## 2 Interactions Between Host Immunity and *M. tuberculosis* During the Course of Infection

**FIGURE 1:**
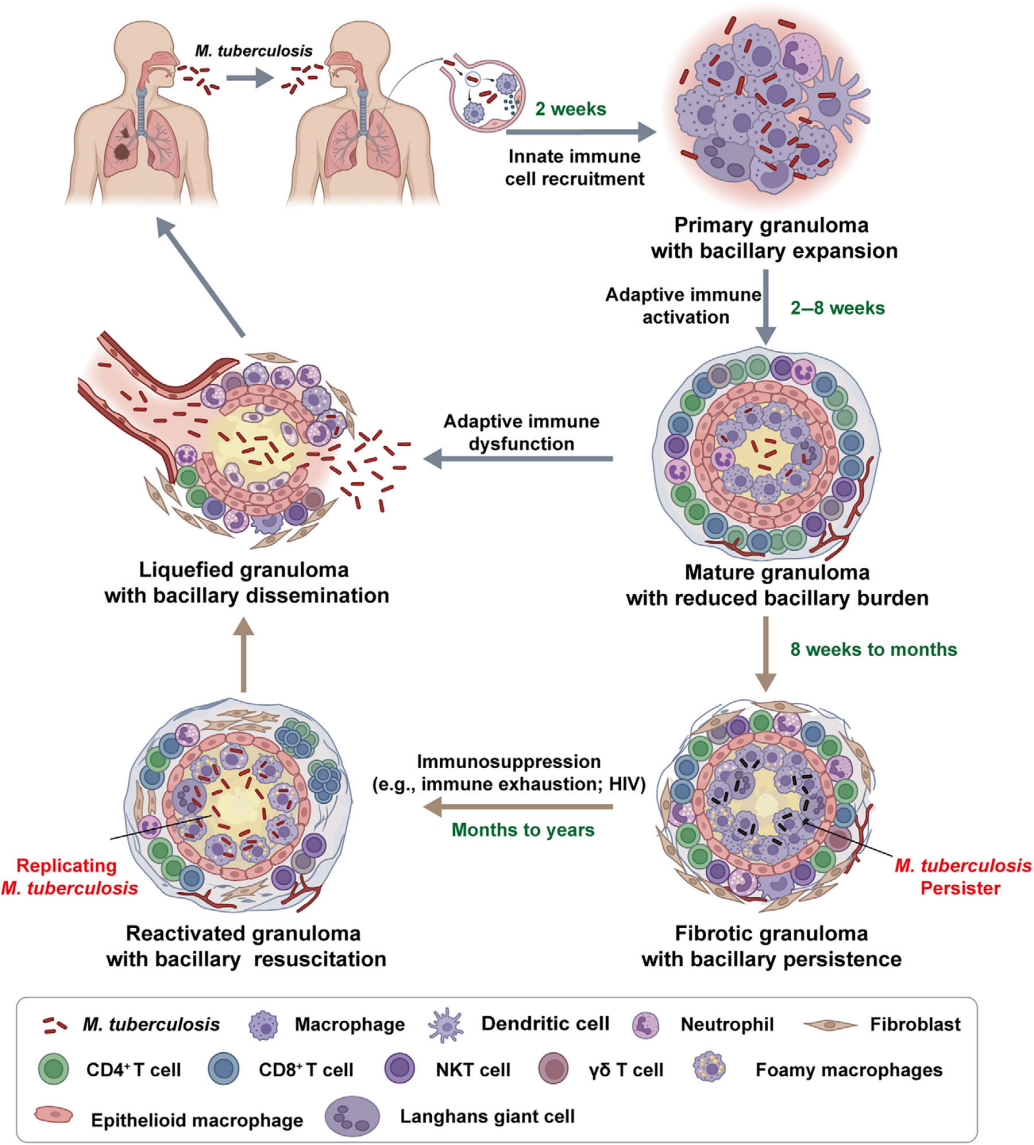
The occurrence and progression of tuberculosis granuloma. Following *M. tuberculosis* infection, alveolar macrophages phagocytose the pathogen and recruit other innate immune cells, such as dendritic cells and neutrophils, leading to the formation of early granulomas accompanied by bacterial expansion at approximately 2 weeks. Subsequently, between weeks 2 and 8, adaptive immunity is activated, and infiltrating T cells promote the differentiation of macrophages into epithelioid cells and multinucleated giant cells, thereby forming mature granulomas capable of restricting the bacterial load. From 8 weeks to several months postinfection, the development of fibrosis in approximately 90% of infectious lesions restricts pathogen dissemination, driving the bacteria to alter their metabolic state and form persisters that survive long‐term within the granuloma. Months to years later, when the host experiences immunosuppression, such as immune exhaustion, alongside impaired adaptive immunity, these persister bacteria can resuscitate, further causing liquefactive necrosis and structural destruction of the granuloma, which ultimately leads to secondary bacterial dissemination and cavity formation.

The pathogenesis of tuberculosis is a dynamic process shaped by continuous interactions between *M. tuberculosis* and the host immune system. From early granuloma formation to the development of tissue‐destructive disease (Figure 1), each stage reflects a battle between host defenses and bacterial immune evasions. Within tuberculosis granulomas, host immunity attempts to constrain bacillary replication and preserve tissue architecture, whereas *M. tuberculosis* subverts and remodels the local immune microenvironment to promote its survival, persistence, and transmission. On this basis, the following section discusses these host–pathogen interactions across four interconnected stages of infection.

### 2.1 Recruitment of Innate Immune Cells, *M. tuberculosis* Evasion, and Early Granuloma Formation

**FIGURE 2:**
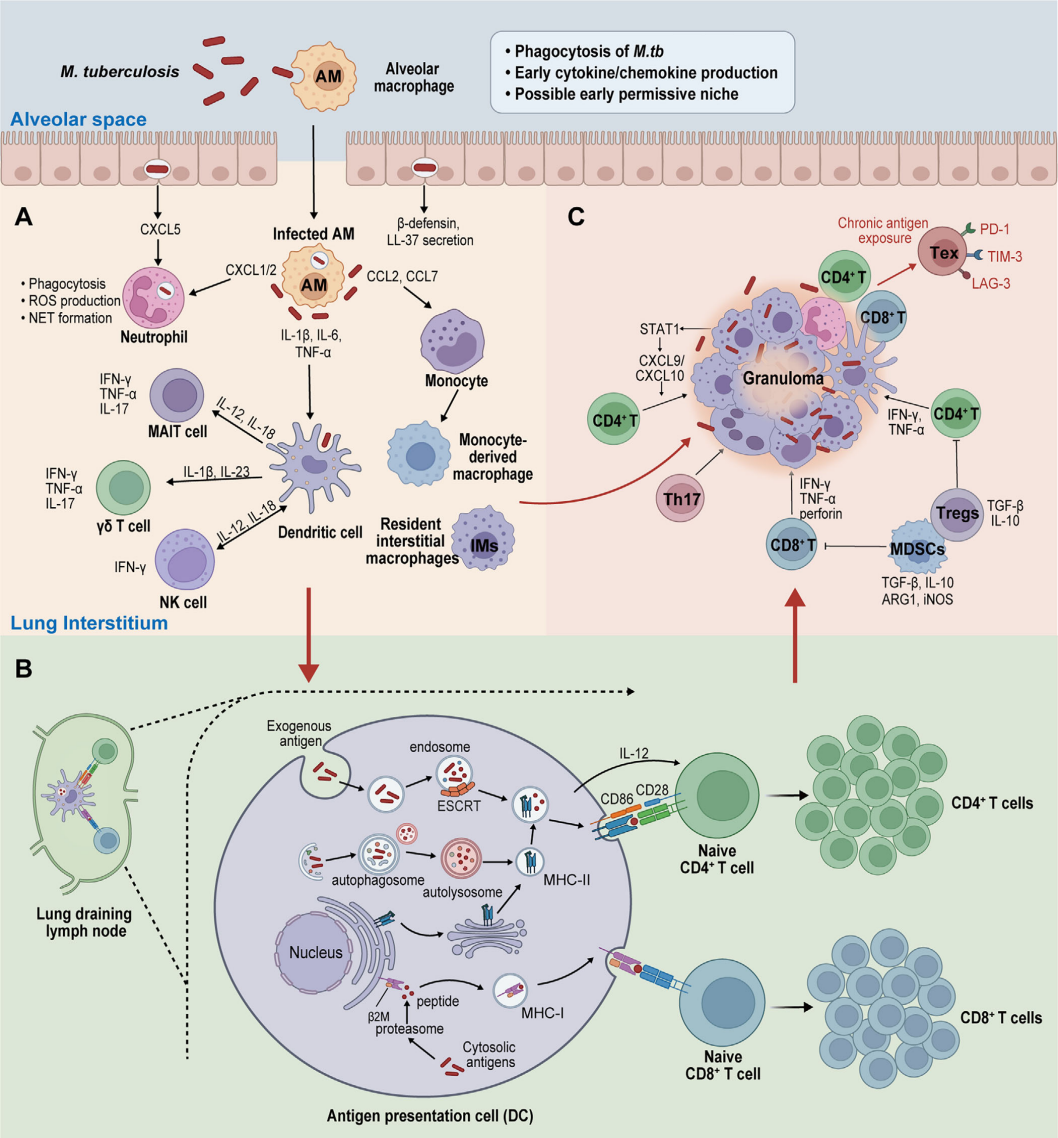
Host innate and adaptive immune responses during *M. tuberculosis* infection. (A) Early innate immune response. After entering the alveolar space, *M. tuberculosis* is phagocytosed by alveolar macrophages (AMs) and can also interact with airway epithelial cells. Infected epithelial cells and macrophages secrete cytokines and chemokines, including GM‐CSF, CXCL5, CXCL1/2, CCL2, and CCL7, which recruit neutrophils, monocytes, dendritic cells (DCs), natural killer (NK) cells, mucosal‐associated invariant T (MAIT) cells, and γδ T cells to the infected interstitium. (B) Antigen presentation and T‐cell priming. Infected DCs migrate to lung‐draining lymph nodes, where they process and present mycobacterial antigens through MHC‐I and MHC‐II pathways to prime naïve CD8^+^ and CD4^+^ T cells, respectively. (C) Adaptive immune response. After activation in the draining lymph nodes, active antigen‐specific T cells migrate back to the infected lung. However, regulatory T cells(Tregs) and myeloid‐derived suppressor cells (MDSCs) suppress Th1 and CD8^+^ T‐cell responses, and persistent antigen exposure further drives T‐cell exhaustion, collectively facilitating bacterial persistence within granulomatous lesions.

During the early stage of infection, *M. tuberculosis* is initially phagocytosed predominantly by AMs, although pulmonary epithelial cells may also become infected. Subsequently, infected AMs migrate from the alveolar lumen into the lung interstitium, where additional innate immune cell populations, including monocyte‐derived phagocytes, neutrophils, DCs, NK cells, and nonclassical T cells, are rapidly recruited (Figure 2A). Together, these cells cooperate to restrict bacterial expansion through phagocytosis, cytokine secretion, cytotoxic activity, and the initiation of adaptive immune responses. Nevertheless, *M. tuberculosis* often evades sterilizing clearance, thereby facilitating the formation of nascent granulomas. In this section, the interactions between *M. tuberculosis* and innate immune cells during this early stage are discussed.

#### 2.1.1 Macrophages

##### 2.1.1.1 The Heterogeneity of Macrophages

Macrophages are the frontline cells of innate defense in the lung and also play a central role in the pathogenesis of *M. tuberculosis* infection. According to their ontogeny, lung macrophages are broadly classified into tissue‐resident AMs and monocyte‐derived interstitial macrophages (IMs) [12].

AMs are the predominant cell type infected during the first 2 weeks of infection. As sentinels of the alveolar space, they recognize, engulf, and initially attempt to restrict *M. tuberculosis* growth through the lysosomal enzymes release and reactive oxygen species (ROS) production [18]. However, accumulating evidence suggests that *M. tuberculosis* is not always efficiently controlled within AMs. In mouse models, selective depletion of AMs has been shown to reduce the pulmonary bacterial burden, indicating that during the earliest stage of infection, AMs may serve more as a relatively permissive niche for bacterial replication than as highly effective bactericidal cells [11]. Mechanistically, Rothchild et al. demonstrated that *M. tuberculosis* infection induces an NRF2‐centered antioxidant transcriptional program in AMs, thereby compromising early bacterial control [19]. Nevertheless, AMs should not be regarded as a functionally uniform population with fixed properties. As infection progresses and adaptive immunity develops, some AM subsets may acquire a more potent antimicrobial phenotype under the influence of signals such as IFN‐γ [20]. Therefore, the role of AMs in host defense against *M. tuberculosis* is clearly stage‐specific and state‐dependent.

After approximately 2 weeks of infection, infected AMs migrate from the alveolar space into the lung interstitium, where *M. tuberculosis* can subsequently infect monocyte‐derived IMs. Traditionally, IMs have been considered more restrictive to *M. tuberculosis* than AMs. Supporting this view, depletion of circulating monocytes by intravenous clodronate administration in mice leads to an increased pulmonary bacterial burden, suggesting that monocyte‐derived macrophages contribute to host protection during infection [21]. In addition, transcriptomic analyses of human monocyte‐derived macrophages have shown that these cells mount a stronger inflammatory response to *M. tuberculosis* than AMs [22]. However, recent single‐cell RNA sequencing studies indicate that pulmonary IMs are not a homogeneous population, but instead comprise multiple subsets with distinct differentiation states, activation profiles, and antimicrobial capacities [23]. Therefore, the statement that “IMs are more restrictive than AMs” is an oversimplification.

Taken together, during the early stage of pulmonary tuberculosis, AMs are the first population to encounter *M. tuberculosis*, but they appear to exert limited control over bacterial replication. By contrast, monocyte‐derived IMs are generally considered to have greater early antimycobacterial potential. Nevertheless, both AMs and IMs consist of heterogeneous subsets, and these subsets differ substantially in their ability to control infection. These differences in antimicrobial capacity among distinct macrophage populations may be closely associated with their underlying metabolic phenotypes, as discussed in more detail below.

##### 2.1.1.2 Mechanisms of Macrophage‐Mediated Control of *M. tuberculosis* and Bacterial Evasion Strategies

**FIGURE 3:**
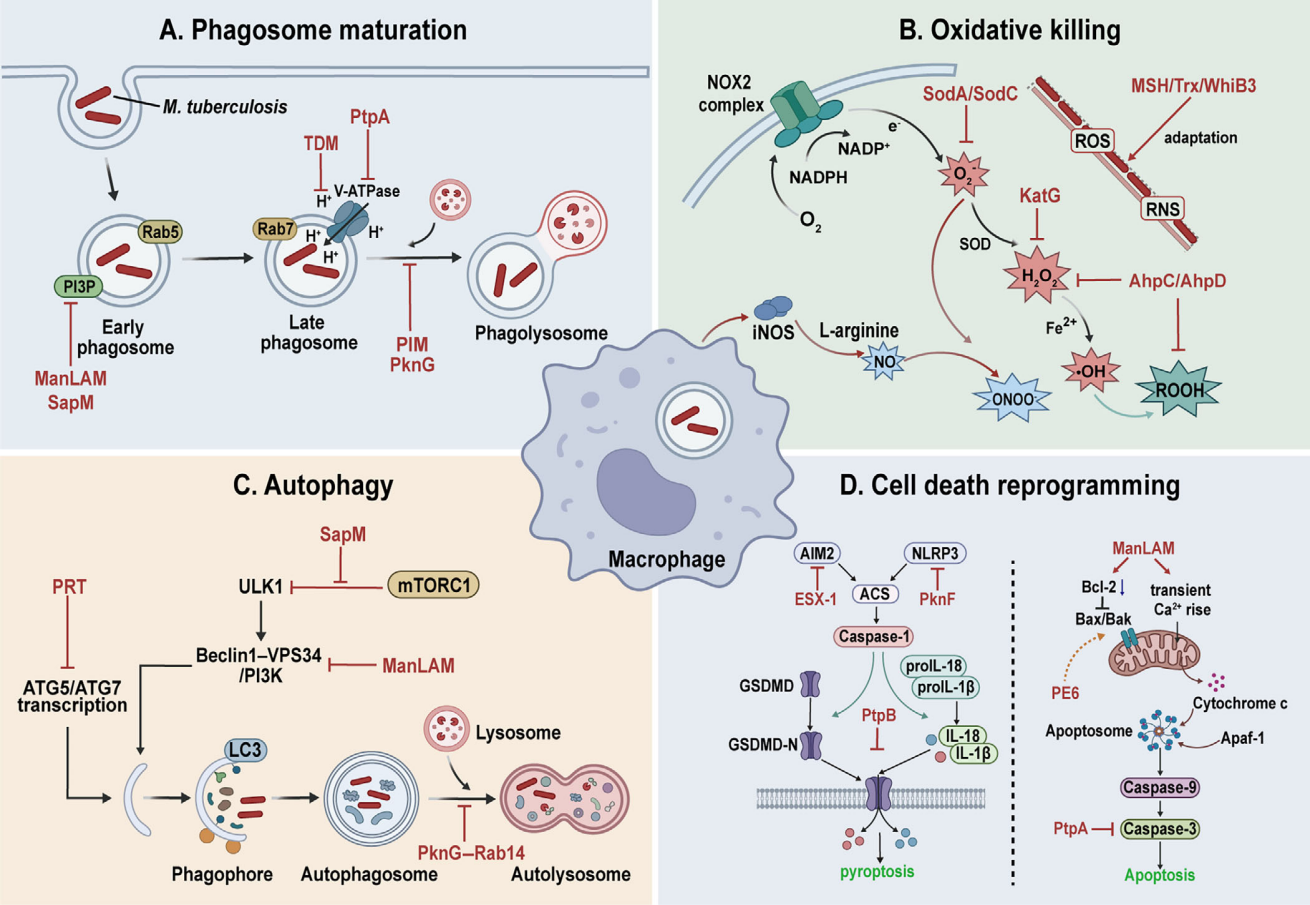
Macrophage antimicrobial mechanisms and immune evasion strategies of *M. tuberculosis*. (A) Phagosome maturation. ManLAM and SapM impair PI3P‐dependent early phagosomal signaling, whereas TDM modulates phagosomal acidification. In addition, PtpA inhibits V‐ATPase recruitment and activity, and PIM and PknG further disrupt late phagosomal trafficking, thereby preventing efficient phagosome–lysosome fusion and bacterial degradation. (B) Oxidative killing. Macrophages generate reactive oxygen species (ROS) through the NOX2 complex and reactive nitrogen species (RNS) through inducible nitric oxide synthase (iNOS). *M. tuberculosis* counteracts these antimicrobial stresses by using antioxidant and redox‐buffering systems, including SodA/SodC, KatG, AhpC/AhpD, and the mycothiol–thioredoxin–WhiB3 network, which detoxify superoxide, hydrogen peroxide, organic peroxides, and RNS‐derived intermediates, thereby promoting bacterial survival under oxidative and nitrosative stress. (C) Autophagy. Autophagy contributes to intracellular bacterial clearance by delivering bacilli‐containing compartments to lysosomes. *M. tuberculosis* suppresses this process at multiple steps: SapM inhibits mTORC1–ULK1‐associated autophagy signaling, ManLAM interferes with the beclin‐1–VPS34/PI3K complex, PRT suppresses ATG5/ATG7 transcription, and PknG‐Rab14 signaling impairs autophagosome maturation and autolysosome formation. (D) Cell death reprogramming. *M. tuberculosis* modulates macrophage cell death pathways to create a niche favorable for intracellular persistence. ESX‐1 can engage inflammasome‐related pathways, whereas PknF and PtpB interfere with NLRP3/caspase‐1/GSDMD‐mediated pyroptosis and IL‐1β maturation. In parallel, ManLAM, PE6, and PtpA modulate mitochondrial apoptotic signaling, thereby inhibiting apoptosis.

Macrophages mount a multifaceted intracellular defense program against *M. tuberculosis* through phagolysosomal killing, oxidative and nitrosative stress [24], autophagy [25, 26], and programmed cell death [27, 28] (Figure 3). Nevertheless, *M. tuberculosis* has evolved a sophisticated repertoire of countermeasures that enables it to circumvent macrophage antimicrobial mechanisms and establish an intracellular niche (Figure 3).

First, macrophages eliminate invading pathogens by forming phagolysosomes in which engulfed microbes are degraded within an acidic and hydrolytic compartment. However, *M. tuberculosis* disrupts this bactericidal process by interfering with phagosome maturation and acidification, and by blocking phagosome–lysosome fusion. For example, the mycobacterial cell wall glycolipid mannose‐capped lipoarabinomannan (ManLAM) inhibits calcium/calmodulin signaling and Rab5‐mediated PI3K recruitment, thereby blocking de novo synthesis of phosphatidylinositol 3‐phosphate (PI3P) [29]. In parallel, the secreted acid phosphatase SapM directly hydrolyzes pre‐existing PI3P on the phagosomal membrane [30], further impairing phagosome maturation. In addition, the secreted phosphatase PtpA directly interacts with vacuolar ATPase (V‐ATPase), suppressing proton pump activity and thereby increasing phagosomal pH [31]. The major mycobacterial cell wall lipid trehalose 6,6′‐dimycolate (TDM) also interfere with macrophage pH regulatory mechanisms and delay phagosomal acidification [32]. Moreover, phosphatidylinositol mannosides (PIMs) disrupt the delivery of lysosomal components to phagosomes through host PI3K‐ and EEA1‐dependent pathways [33]. The *M. tuberculosis* serine/threonine protein kinase PknG further interferes with phagosome–lysosome fusion by targeting host Rab7‐interacting lysosomal protein (RILP) and inhibiting recruitment of the homotypic fusion and vacuole protein sorting (HOPS) complex, thereby preventing effective lysosomal docking to phagosomes and ultimately blocking phagolysosome formation [34].

Second, macrophage‐derived ROS and reactive nitrogen species (RNS) impose potent antimicrobial pressure on *M. tuberculosis* by damaging macromolecules and redox‐sensitive metabolic processes [24].

In response, *M. tuberculosis* counteracts this stress through a hierarchically organized defense system. Its lipid‐dense cell envelope provides as a first‐line permeability barrier [35, 36], whereas enzymatic scavengers including KatG [37], SodA/SodC [38, 39], and the AhpC/AhpD‐linked peroxidase machinery detoxify superoxide, peroxides, and peroxynitrite [40, 41]. In parallel, thioredoxin (Trx)‐ and mycothiol (MSH)‐dependent redox buffering systems preserve intrabacterial redox poise, and redox‐sensing regulators such as WhiB3 reprogram bacterial physiology to favor persistence under immune pressure [42, 43, 44].

In the regulation of autophagy, under normal conditions, infection‐ and stress‐related signals normally relieve the inhibitory effect of mTORC1 on the autophagy‐initiating kinase ULK1, thereby triggering the recruitment of autophagy‐related proteins, autophagosome formation, and subsequent fusion with lysosomes. However, *M. tuberculosis* can suppress this process at multiple levels. First, at the stage of autophagy initiation, ManLAM may impair autophagy initiation by interfering with the VPS34/PI3P axis, the Ca^2^^+^–mTOR/ULK1 pathway, and beclin‐1‐associated regulatory pathways [45]. Second, during autophagic flux, pknG inhibits the maturation of autophagosomes into autolysosomes and ultimately disrupts autophagic flux by interacting with the host small GTPase Rab14 [46]. In addition, sapM can inhibit autophagy initiation through a raptor–mTORC1‐dependent mechanism, whereas phosphoribosyltransferase (PRT) suppresses the transcription of autophagy‐related genes, such as atg5 and atg7, through epigenetic remodeling [47, 48, 49].

Finally, when the aforementioned intracellular restriction mechanisms are insufficient to eliminate intracellular *M. tuberculosis*, macrophages initiate programmed cell death, including apoptosis and pyroptosis. However, *M. tuberculosis* does not merely passively endure these host defenses; rather, it actively modulates different cell death programs at multiple checkpoints through a variety of effector molecules. Previous studies have shown that ManLAM can impair the mitochondria‐dependent apoptotic program by interfering with Ca^2^^+^‐dependent signaling, inhibiting the caspase cascade, and enhancing antiapoptotic signals such as bcl‐2 [50]. PtpA can further restrict caspase‐3 activation [51]. In contrast, certain *M. tuberculosis* virulence proteins, such as PE6 encoded by Rv0335c, can induce a Bax/Bak‐associated mitochondrial pathway [52], suggesting that the regulation of host apoptosis by *M. tuberculosis* is stage‐specific and context‐dependent rather than absolutely inhibitory. *M. tuberculosis* also modulate macrophage pyroptosis. For example, PknF suppresses activation of the NLRP3 inflammasome [53]; PtpB inhibits GSDMD‐N pore formation by altering the phospholipid composition of the plasma membrane, thereby blocking pyroptotic execution [54]. In addition, *M. tuberculosis* can suppress AIM2 inflammasome‐mediated pyroptosis through the ESX‐1 secretion system [55]. Taken together, *M. tuberculosis* reshapes host cell death programs by suppressing protective apoptosis and inflammasome‐dependent pyroptosis, thereby prolonging intracellular survival and attenuating host inflammatory defense.

#### 2.1.2 Respiratory Epithelial Cells

Respiratory epithelial cells constitute an important early barrier against *M. tuberculosis* by providing both physical protection and innate antimicrobial activity. In particular, alveolar epithelial cells produce antimicrobial peptides, including β‐defensins and LL‐37, which contribute to local antimycobacterial defense [56, 57]. In addition, epithelial cells secrete cytokines and chemokines that shape the early immune response by recruiting and activating neutrophils, monocytes, macrophages, and lymphocytes [58]. For example, epithelial‐derived chemokines such as CXCL5 promote neutrophil recruitment, whereas mediators including GM‐CSF help reinforce local antimycobacterial immunity [59, 60].

However, alveolar epithelial cells may also be exploited by *M. tuberculosis* during early infection. Although they are not professional phagocytes, they express surface molecules that facilitate bacterial adhesion and entry. One representative example is heparin‐binding hemagglutinin adhesin (HBHA), which binds epithelial heparan sulfate proteoglycans and promotes bacterial attachment; interactions with epithelial integrins have also been implicated in internalization and transcytosis of bacilli across the epithelial barrier [61, 62, 63, 64]. These observations suggest that respiratory epithelial cells can serve as a portal for invasion and possibly for dissemination beyond the alveolar space.

#### 2.1.3 NK Cells

NK cells are an important resident lymphocyte subset in the lungs and exert protective effects primarily by killing infected host cells [65, 66]. Upon stimulation with *M. tuberculosis* antigens, NK cells upregulate natural cytotoxicity receptors such as NKp30, NKp44, and NKp46. These receptors recognize specific ligands on the surface of infected cells, triggering the perforin–granzyme pathway and inducing apoptosis [67, 68]. For example, NKp44 can recognize lipoarabinomannan (LAM) in the *M. tuberculosis* cell wall, mediating a nonphagocytic clearance mechanism through Toll‐like receptor 2 (TLR2) signaling [65]. NK cells may also detect *M. tuberculosis*‐infected cells through a “missing‐self” mechanism [67]. Under normal conditions, inhibitory receptors such as KIRs and NKG2A recognize MHC Class I molecules on healthy cells and suppress NK‐cell activation. When MHC Class I expression is reduced on infected or stressed cells, this inhibitory signaling is weakened, thereby facilitating NK‐cell‐mediated killing [67, 69].

Interestingly, NK cells can develop memory‑like properties after mycobacterial antigen exposure. In mouse models, BCG‑induced memory‐like NK cells continuously produce IFN‑γ and improve pathogen clearance during secondary infection [70, 71]. Consistent with this, clinical studies have shown that after BCG vaccination, the proportion of IFN‑γ producing NK cell subsets in infant cord blood is positively correlated with vaccine protection [72], supporting a potential role for NK cells in vaccine‐induced protection. However, NK cells from patients with pulmonary tuberculosis upregulate inhibitory receptors such as PD‐1, and increased PD‐1 signaling is associated with reduced IFN‐γ production and impaired degranulation; blockade of the PD‐1 pathway can partially restore these effector functions [73]. Similarly, TIM‐3 expression is elevated on NK cells from patients with active tuberculosis, and its expression level is negatively correlated with IFN‐γ production [74, 75]. Together, these findings suggest that NK cells may develop memory‐like protective features after vaccination or antigen priming, whereas prolonged antigenic stimulation during active disease may drive an exhaustion‐like state that favors bacterial persistence.

#### 2.1.4 Neutrophils

Neutrophils contribute to the clearance of *M. tuberculosis* through a network of mechanisms including chemotaxis, phagocytosis, ROS production, protease release, and formation of NETs [76]. After *M. tuberculosis* invasion, lung tissue rapidly releases chemokines such as CXCL8 (IL‐8) and CXCL1, guiding neutrophils from the bloodstream to sites of infection [77]. After *M. tuberculosis* is phagocytosed, neutrophil phagosomes fuse with granule membranes to create phagolysosomes with strong bactericidal activity. During this process, the NADPH oxidase complex generates superoxide and other ROS, while granule proteases are released into the phagolysosome to kill bacteria through oxidative and enzymatic damage [78]. The oxidative burst reaction triggered by neutrophils when phagocytizing *M. tuberculosis* is significantly stronger than that of macrophages [79]. For extracellular *M. tuberculosis*, neutrophils can release NETs, which are web‐like structures composed of DNA and granule proteins that help entrap bacilli [80].

Similar to *M. tuberculosis* immune evasion strategies in macrophages, *M. tuberculosis* can also reduce neutrophil‐mediated killing by inhibiting granule‐phagosome fusion and enhancing redox buffering capacity, although the underlying molecular mechanisms appear to be more cell type‐specific and remain less well defined. In the context of postphagocytic killing, virulence factors such as ESAT‐6, PknG, SapM, PtpA, and Zmp1 have been shown to inhibit phagosome maturation or phagolysosome fusion in macrophages [81, 82], but their roles and molecular targets in neutrophils have not yet been fully elucidated. Available evidence indicates that *M. tuberculosis* inhibit activation of the Src family kinase Hck and disrupt F‐actin reorganization, thereby blocking granule–phagosome fusion and limiting the delivery of granule‐derived antimicrobial components to bacteria‐containing phagosomes [83]. In addition, the neutrophil MPO–H_2_O_2_–Cl^−^ system generates hypochlorous acid (HOCl), which represents one of its most neutrophil‐derived bactericidal mechanisms. To counter this oxidative stress, mycobacteria rely on redox buffering systems such as Trx and MSH to maintain intrabacterial redox homeostasis [84, 85]. They can also secrete low‐molecular‐weight sulfur‐containing metabolites, including hydrogen sulfide (H_2_S) and ergothioneine (EGT), to directly neutralize HOCl and thereby reduce oxidative damage [86, 87].

#### 2.1.5 Dendritic Cells

DCs form extensive and dense networks throughout the airway mucosa and represent a major population of antigen‐presenting cells (APCs) capable of presenting *M. tuberculosis‐*specific antigens to T cells. Upon sensing and phagocytosing *M. tuberculosis*‐derived Ags, DCs undergo a maturation process, migrate to draining lymph nodes (dLNs) and present antigens to T lymphocytes [88, 89, 90]. After the adaptive immune response has been initiated, DCs dynamically traffic in and out of granulomas to sustain systemic T‐cell responses [91, 92].

DC function is not unilateral; rather, it is dynamically regulated through bidirectional interactions with multiple immune and nonimmune cell populations. For example, NK cells produce IFN‐γ, which promotes DC maturation and enhances the differentiation of naïve T cells into Th1 effector cells [93, 94]. In addition, BCG‐infected neutrophils have been shown to reduce IL‐10 secretion by human DCs and to enhance DC‐mediated antigen presentation, thereby promoting CD4^+^ and CD8^+^ T‐cell proliferation. Moreover, recent evidence indicates that *M. tuberculosis*‐infected AEC‐II indirectly induce DC maturation by negatively regulating hypoxia‐inducible factor‐1α (HIF‐1α)‐dependent NOS2 expression and reprogramming DC metabolic pathways [95].

DCs are also targeted by *M. tuberculosis* as part of its immune evasion strategy. For example, *M. tuberculosis* cell wall components, such as ManLAM and Di‐O‐acyl trehalose engage C‐type lectin receptors, including DC‐SIGN, DCIR, Dectin‐2, and Mincle, and frequently skew DCs toward a tolerogenic phenotype characterized by reduced expression of costimulatory molecules and MHC Class II, diminished IL‐12 production, and enhanced secretion of IL‐10 and increased indoleamine 2,3‐dioxygenase (IDO) expression, ultimately promoting FOXP3^+^ regulatory T cell expansion and suboptimal Th1 immunity [96, 97, 98, 99]. In parallel, multiple *M. tuberculosis*‐derived proteins‐including ESAT‐6, Hip1, GroEL2, Zmp1, PE‐PGRS47, and Acr‐1‐interfere with DC maturation and antigen presentation by disrupting phagosome maturation, inhibiting autophagosome–lysosome fusion, or impairing endosomal sorting complexes, thereby limiting efficient MHC Class II‐restricted antigen presentation and Th1/Th17 polarization [100, 101, 102, 103, 104]. Emerging studies further indicate that *M. tuberculosis*‐driven metabolic reprogramming of DCs contributes to impaired maturation and immunogenicity, linking immunometabolic dysregulation to defective T cell priming during tuberculosis [105, 106, 107].

#### 2.1.6 Nonclassical T Cells

Among nonclassical T cells, γδ T cells, mucosal‐associated invariant T (MAIT) cells and invariant NK T (iNKT) cells play important roles in anti‐*M. tuberculosis* immunity through unique antigen‐recognition patterns and effector functions. During the early stages of infection, Vγ9Vδ2 γδ T cells rapidly respond, recognizing phosphorylated antigens and undergoing rapid activation [108]. Consistent with this, recent T‐cell receptor (TCR) sequencing studies have shown that the CDR3 region of Vγ9Vδ2 T‐cell subsets in tuberculosis patients exhibits oligoclonal enrichment, supporting antigen‐driven clonal expansion of this population [109]. Once activated, γδ T cells produce cytokines such as IFN‐γ, TNF‐α, IL‐17, and IL‐10, thereby enhancing macrophage‐mediated bacterial killing and also shaping the inflammatory environment to control *M. tuberculosis* [110, 111]. Evidence from rhesus macaque infection model further supports their early protective role, as γδ T cells expand rapidly in the airways within 24 h after exposure to attenuated *Mycobacterium bovis* (M. bovis) BCG [112]. Additionally, in heterologous vaccine studies using Listeria monocytogenes, vaccine‐induced Vγ9Vδ2 T cells expanded in lung tissue and limited *M. tuberculosis* spread [113]. Beyond their direct effector functions, activated Vγ9Vδ2 T cells can also acquire APC‐like properties and promote the proliferation and activation of αβ T cells [114].

MAIT cells are another important component in the early immune response. They are widely distributed in mucosal barrier tissues and peripheral blood and specifically recognize microbial metabolites derived from riboflavin biosynthesis that are presented by the MHC Class I‐related molecule MR1 [115]. In early M. bovis BCG infection, MR1‐deficient mice exhibit significantly higher pulmonary bacterial burdens than wild‐type mice [116]. Similarly, after infection with *M. tuberculosis* or BCG, Vα19 transgenic mice showed significantly lower lung bacterial loads than control mice [117]. In rhesus macaque models, BCG vaccination or aerosol *M. tuberculosis* infection induced antigen‐specific MAIT‐cell activation, with high frequencies of granzyme B‐producing MAIT cells detected both in peripheral blood and at sites of infection [118]. Together, these findings support a protective role for MAIT cells during the early stage of mycobacterial infection. However, active tuberculosis patients show “peripheral depletion and local enrichment” of MAIT cells, which secrete TNF‑α to resist *M. tuberculosis* [119].

iNKT cells recognize lipid and glycolipid antigens presented by the nonpolymorphic CD1d molecule and serve as a bridge between innate and adaptive immunity. Upon activation, iNKT cells rapidly produce cytokines (e.g., IFN‐γ, IL‐4) and influence the functions of multiple immune cell types, including macrophages, DCs, and conventional T cells [120]. Studies using murine models have shown that iNKT cells can restrict *M. tuberculosis* replication both in vitro and in vivo. In these systems, iNKT cells produce IFN‐γ following activation by infected macrophages and are capable of directly limiting intracellular bacterial growth in a CD1d‐dependent manner, even in the absence of classical adaptive immune responses [121]. Another effector function of iNKT cells is the production of granulocyte–macrophage colony‐stimulating factor (GM‐CSF) [122]. GM‐CSF enhances antimicrobial activity by promoting the function of other immune cells such as DCs and T cells, highlighting the role of iNKT cells in orchestrating coordinated immunity against *M. tuberculosis*. Studies of latent *M. tuberculosis* infection suggest that iNKT cells (often alongside other innate T cells like MAIT cells) may be more frequent in individuals with controlled infection than in those with active disease. This observation supports a role in containment of latent infection, though their functional contributions remain under active investigation [123]. These animal and human studies suggest that iNKT cells contribute to protective immunity against tuberculosis infection.

Overall, during the early stage of *M. tuberculosis* infection, multiple innate immune cell populations are rapidly recruited to sites of infection and cooperate to restrict bacterial expansion. As infection progresses, these early cellular aggregates gradually become spatially organized, laying the foundation for early granuloma formation, which serve as an initial host attempt to contain infected cells and localize bacilli within a structured inflammatory niche. However, *M. tuberculosis* is not a passive target of host defense; rather, it has evolved a range of finely tuned immune evasion strategies that remodel host responses at multiple critical checkpoints through both cell‐wall lipids and secreted effector molecules. Although innate immune cells are indispensable for antituberculosis immunity, the fact that approximately one‐quarter of the global population is infected with *M. tuberculosis* indicates that innate immunity alone is often insufficient to achieve stable and complete bacterial clearance. Given the marked heterogeneity, plasticity, and trained immunity potential of innate immune cells, future strategies may, on the one hand, exploit strategies to enhance the antimycobacterial capacity of permissive cell subsets and shift the balance of infection toward a more restrictive state; on the other hand, selected mycobacterial components or their derivatives may be explored as inducers of trained immunity as part of novel vaccine strategies to strengthen early protective responses against *M. tuberculosis*.

### 2.2 Adaptive Immune Activation, *M. tuberculosis* Control, and Granuloma Organization

As infection progresses, DCs and other APCs transport *M. tuberculosis* antigens to dLNs, where they prime antigen‐specific T‐cell responses. This process leads to the activation of adaptive immune populations, particularly CD4^+^ Th1 cells, CD8^+^ T cells (Figure 2B,C). At the same time, these adaptive immune cells contribute to the structural organization of the granulomas by coordinating the differentiation, transformation, and spatial arrangement of macrophages and stromal elements (Figure 1). These events help transform early inflammatory lesions into more organized granulomatous structures that limit bacterial spread and establish a state of partial immune control. The activation of adaptive immunity, *M. tuberculosis* evasion, and their contribution to granuloma organization are discussed in this section.

#### 2.2.1 T Cells

T cells are central mediators of adaptive immunity against *M. tuberculosis*, and many current tuberculosis vaccine strategies are designed to elicit durable T‐cell responses [124]. Evidence from both human disease and animal models has clearly demonstrated that T cells are indispensable for controlling primary infection, limiting bacterial replication, and organizing protective granulomatous responses [125, 126]. Nevertheless, T‐cell immunity rarely achieves sterilizing clearance, because its protective effects are constrained by antigen availability and *M. tuberculosis*‐mediated immune evasion.

##### 2.2.1.1 CD4^+^ T Cells

CD4^+^ T cells play a central role in the adaptive immune response against tuberculosis. Their importance is strongly supported by the markedly increased susceptibility to active tuberculosis in individuals with HIV infection and CD4^+^ T‐cell depletion [127]. Following priming by APCs, naïve CD4^+^ T cells can differentiate into several functional subsets, with Th1 and Th17 cells being especially important in tuberculosis immunity.

Th1 cells are the best‐established protective CD4^+^ T‐cell subset in tuberculosis. They produce IFN‐γ, TNF‐α, and IL‐2, which together enhance antimycobacterial immunity [128]. IFN‐γ is a key activator of infected macrophages, promoting phagolysosomal maturation, antimicrobial gene expression, and nitric oxide (NO) production through induction of inducible NO synthase (iNOS), particularly in murine models [129, 130]. The clinical importance of TNF‐α is underscored by the increased risk of tuberculosis reactivation during anti‐TNF therapy [131]; it synergizes with IFN‐γ to maintain macrophage activation and contributes to granuloma formation and stability [132]. IL‐2 mainly supports T‐cell proliferation, survival, and memory formation rather than acting as a major direct antimycobacterial effector [133, 134]. Thus, protective Th1 immunity in tuberculosis is better understood as a coordinated macrophage‐activating program driven primarily by IFN‐γ and TNF‐α, with IL‐2 supporting the expansion and persistence of antigen‐specific T cells.

Th17 cells primarily produce effector cytokines such as IL‐17A, IL‐17F, and IL‐22 [135, 136], which stimulate pulmonary epithelial and stromal cells to produce chemokines and granulopoietic factors, thereby promoting the recruitment of neutrophils, monocytes, and other inflammatory cells and helping establish an early local defense environment in the lung [137, 138]. Early studies showed that IL‐17 and IL‐22 responses are induced during human mycobacterial infection [139], and that BCG vaccination induced IL‐17‐producing CD4^+^ T cells contributing to bacterial control [140]. However, subsequent studies have shown that the role of Th17 cells during primary *M. tuberculosis* infection is context‐dependent and sometimes conflicting. For example, Khader and colleagues found that IL‐17 was dispensable for primary immunity against low‐dose infection with a laboratory *M. tuberculosis* strain in mice [141]. In contrast, Gopal et al. demonstrated that IL‐17 contributes to protective immunity against infection with the hypervirulent HN878 strain [142]. In humans, the role of Th17/IL‐17 responses in tuberculosis has largely been inferred, but these studies have also yielded inconsistent results [139, 143]. Recent study has shown that *M. tuberculosis*‐reactive Th17 cells are not a uniform population, suggesting that future studies should move beyond measuring total Th17‐cell frequencies or bulk IL‐17 levels and instead define which Th17 subsets are present, where they localize.

##### 2.2.1.2 CD8^+^ T Cells

Although *M. tuberculosis* is mainly confined to phagosomes, a compartment that is not readily accessible to the classical MHC Class I presentation pathway, CD8^+^ T‐cell priming can still occur through cross‐presentation. Schaible et al. showed that DCs acquire mycobacterial antigens from apoptotic infected cells and cross‐present them to CD8^+^ T cells, providing a key mechanism for CD8^+^ T‐cell activation during tuberculosis [144]. In murine tuberculosis models, loss of CD8^+^ T cells is associated with increased bacterial burdens, whereas adoptive transfer of immune CD8^+^ T cells reduces mycobacterial loads, underscoring their protective role in infection control [145, 146]. Once activated, CD8^+^ T cells exert antimycobacterial effects through perforin‐mediated cytolysis of infected macrophages and, thereby limiting intracellular bacilli [15, 147], and by producing cytokines such as IFN‐γ, IL‐2, and TNF‐α that reinforce antimicrobial immunity [148]. Together, these observations support an important role for CD8^+^ T cells in the early control of *M. tuberculosis* infection.

##### 2.2.1.3 *M. tuberculosis*‐Mediated Delay in T‐Cell Priming and Restriction of T‐Cell Migration

A hallmark of antituberculosis adaptive immunity is the delayed priming of T cells. In humans, *M. tuberculosis*‐specific T‐cell responses are typically not detectable until 2–8 weeks after infection. A major basis for this delay is that naïve T cells can be effectively activated only after bacilli or their antigens are transported to lung‐dLNs, which intrinsically postpones the initiation of adaptive immunity in tuberculosis [149].

*M. tuberculosis* further delays T‐cell priming through multiple mechanisms that impair DC maturation and antigen presentation. First, *M. tuberculosis* can directly interfere with antigen processing and MHC loading. For example, the effector protein EsxH disrupts the function of the endosomal sorting complex required for transport (ESCRT), thereby impairing antigen processing and MHC Class II presentation and weakening effective CD4^+^ T‐cell activation [150]. Second, the secreted virulence factor ESAT‐6 can directly bind β_2_‐microglobulin (β_2_M), preventing its proper assembly with the MHC Class I heavy chain. This disrupts MHC Class I folding and causes retention of the complex in the endoplasmic reticulum, thereby impairing antigen presentation relevant to CD8^+^ T‐cell activation [151]. In addition to directly disrupting MHC presentation pathways, *M. tuberculosis* can also reduce antigen visibility by weakening antigen immunogenicity and suppressing auxiliary antigen‐presentation pathways. The cell envelope‐associated serine protease Hip1 cleaves the chaperone‐like protein GroEL2, generating a poorly immunostimulatory form that compromises DC maturation and antigen presentation [102]. Meanwhile, PE_PGRS47 suppresses host autophagy and thereby weakens MHC Class II‐restricted antigen presentation [101]. The virulence lipid phthiocerol dimycocerosate (PDIM) can also inhibit the expression of CD86 and IL‐12p40 in infected DCs, weakening costimulatory signaling and the Th1‐polarizing cytokine milieu. Consequently, even when naïve T cells encounter antigen, they may still fail to receive sufficient activation signals [152].

In addition, *M. tuberculosis* can reshape the inflammatory microenvironment to limit T‐cell migration. For example, *M. tuberculosis* infection induces IDO1 expression in inflammatory macrophages, leading to kynurenine production, activation of AhR and SOCS3, and reduced CXCL9 and CXCL10 secretion, thereby delaying T‐cell migration to infected lung tissue and postponing adaptive immune activation [153]. *M. tuberculosis*‐specific FOXP3^+^ Treg cells, which expanded in lung‐dLNs after infection can also significantly delay the priming and lung accumulation of effector CD4^+^ and CD8^+^ T cells [154]. Notably, *M*. tuberculosis upregulates Rv1272c under hypoxic conditions, which promotes lecithin uptake, linoleic acid release, and thereby enhancing Treg function [155].

##### 2.2.1.4 *M. tuberculosis*‐Mediated Impairment of T‐Cell Effector Function

In addition to delaying T‐cell priming, *M. tuberculosis* can directly impair T‐cell effector function after these cells enter the infected host environment. Within hypoxic granulomas, *M. tuberculosis* induces the expression of Rv0884c (SerC), a phosphoserine aminotransferase that promotes D‐serine production, which directly inhibits the WDR24–mTORC1–T‐bet pathway in CD8^+^ T cells and thereby reducing IFN‐γ production [156]. These findings suggest that *M. tuberculosis* can actively reprogram the metabolic and signaling state of effector T cells to dampen protective immunity.

Beyond this direct inhibition, accumulating evidence indicates that chronic *M. tuberculosis* infection drives T cells into a hyporesponsive or exhaustion‐like state. In murine tuberculosis, T cells progressively upregulate inhibitory receptors such as PD‐1, TIM‐3, and KLRG‐1, particularly during chronic infection, consistent with functional impairment [157]. TIM‐3^+^ T cells that accumulate during chronic infection show reduced IL‐2 and TNF production and coexpress additional inhibitory receptors, supporting the view that sustained antigen exposure in tuberculosis promotes dysfunctional T‐cell differentiation rather than durable protective effector responses [158].

In addition, the immunotolerant niche within tuberculous granulomas, shaped by suppressive myeloid cells and regulatory T cells, can further impair T‐cell function [159]. For example, Gr1^int^CD11b^+^ myeloid‐derived suppressor cells (MDSCs) highly express Arg1, and Arg1‐dependent arginine deprivation is likely represents an important metabolic basis for T‐cell dysfunction [160, 161]. Tregs constitute another key component of this immunotolerant niche, mainly by secreting suppressive cytokines such as IL‐10 and TGF‐β, thereby inhibiting Th1‐associated IFN‐γ responses and limiting protective antituberculosis immunity [161].

#### 2.2.2 B Lymphocytes

##### 2.2.2.1 The Role of B Cells Against *M. tuberculosis*

B cells have complex roles in tuberculosis immunity, and their responses and functions during *M. tuberculosis* infection remain to fully understood. Compared with the clearly defined role of CD4^+^ T cells in tuberculosis control, the importance of B cells requires further investigation. Recent research shows B cells and their antibody (Ab)‐mediated humoral immunity interact with cellular immune networks to help control intracellular pathogens [162]. In the *M. tuberculosis*‑infected environment, B cells act not only as APCs, interacting with nearby immune cells, but also but also modulate the local inflammatory milieu through cytokine production, thereby contributing to bacterial containment [163, 164, 165]. In mouse models of tuberculosis, B cell knockout or depletion leads to increased susceptibility to tuberculosis [166, 167]. In nonhuman primates, B‑cell depletion results in a significant increase in bacterial load in lung lesions [168]. These animal studies provide direct evidence for B‑cell protection. Single‑cell analysis shows B cells can upregulate PD‑L1 and crosstalk with lung T‑follicular‑helper (Tfh‑like) cells, promoting their maturation and helping control *M. tuberculosis* survival [169]. Moreover, beyond classic B cells, marginal‑zone B cells and other unusual B‑cell subsets increase specifically in lungs and spleens during *M. tuberculosis* infection. These subsets shape the immune microenvironment by secreting characteristic cytokine profiles, exhibiting immunoregulatory functions distinct from those of traditional B cells [170].

At the humoral immunity level, Abs produced by B cells after their differentiation into plasma cells play multidimensional roles in protective immunity against *M. tuberculosis*. In primate studies, intravenous administration of BCG to rhesus monkeys significantly increased IgM levels in lung tissue, and this IgM titer showed a negative correlation with the intra‐pulmonary *M. tuberculosis* burden. Specifically, IgM Abs targeting LAM and Psts‐1 antigens enhanced the phagocytic function of AMs through opsonization, directly inhibiting intracellular survival of *M. tuberculosis* [171]. Similarly, in a mouse model, aerosol *M. tuberculosis* infection experiments using IgM secretion‐deficient mice revealed that these mice had a significantly higher bacterial load in the lungs compared with wild‐type mice, along with markedly increased mortality during the chronic infection phase, accompanied by delayed formation of pulmonary lymphoid follicles and abnormal granuloma structure. However, adoptive transfer of immune serum containing anti‐*M. tuberculosis* Abs partially restored pulmonary granuloma structure and reduced the bacterial load [172]. Beyond opsonization, studies on clinical samples have also shown that Abs in patients with latent tuberculosis can enhance macrophage responses against intracellular *M. tuberculosis* through other mechanisms, including promoting phagolysosome maturation and activating inflammasomes independently of pyroptosis [173]. Furthermore, Fcγ receptors (FcγRs), as key mediators of Ab function, are also indispensable in the immunoregulation of tuberculosis. Studies have found that mice lacking activating FcγRs exhibited enhanced tuberculosis‐associated immunopathology and increased pulmonary bacterial burden, whereas mice lacking inhibitory FcγRs showed significantly reduced lung pathological damage [174].

In summary, B cells take part in tuberculosis control by both contact‐dependent cell interactions and humoral pathways, forming a complex immune regulatory network. Further study of B‐cell subsets and their cooperation with other immune cells could provide novel insights for the development of tuberculosis immunotherapies and vaccines.

##### 2.2.2.2 *M. tuberculosis* Interference with B‐Cell Function and Ab Responses

*M. tuberculosis* can directly interfere with the normal function of B cells, thereby affecting Ab production and efficacy. Studies have shown that LAM binds to TLR2/4 on the surface of B cells, triggering MyD88‐dependent signaling and inducing expansion of regulatory B cells (Bregs). These Breg cells can secrete large amounts of the inhibitory cytokine IL‐10, which suppresses Th1 cell polarization and promotes Th2 cell polarization, thereby weakening host protective immunity against tuberculosis [175]. Furthermore, PtpA can enter host cells and dephosphorylate key kinases, including spleen tyrosine kinase (SYK) and Bruton's tyrosine kinase (BTK) in the B cell receptor (BCR) signaling pathway, inhibiting BCR‐mediated signal transduction [176]. As a result, B cells fail to receive adequate activation signals, limiting plasma‐cell differentiation and Ab secretion.

*M. tuberculosis* may also weaken the immune efficacy of the Abs through specific mechanisms. Studies have shown that *M. tuberculosis* mutants with defects in mycolic acid synthesis, which display markedly reduced cell‐wall hydrophobicity, exhibited increased Ab binding efficiency and enhanced macrophage phagocytosis in mouse infection models [177]. This finding suggests that the highly hydrophobic cell wall of wild‐type *M. tuberculosis* effectively hinders Ab binding. Additionally, the reduced hydrophobic surface of the *M. tuberculosis* cell wall decreases the deposition of complement proteins, thus diminishing complement‐mediated opsonization and bacterial killing [178]. *M. tuberculosis* may also undergo antigenic variation, altering surface‐antigen structure so that pre‐existing Abs fail to recognize the modified antigens, compromising Ab‐mediated neutralization. This facilitates bacterial survival and replication in the host [179]. Clinically, active tuberculosis patients have significantly lower serum IgG against the Rv1733c antigen compared with those with latent infection, possibly reflecting antigenic‐variation‐induced loss of Ab recognition [180].

#### 2.2.3 Transformation of Macrophages Into Epithelioid Cells and Langhans Giant Cells and Organization of Granuloma

As adaptive immunity becomes progressively established, continuously recruited and activated macrophages within lesion undergo marked morphological and functional reprogramming. These cells gradually differentiating into epithelioid cells and Langhans giant cells (LGCs), thereby driving the transition of granulomas from early loose inflammatory cell aggregates into more organized structural units.

Epithelioid cells are regarded as a hallmark component of tuberculous granulomas. They are not merely “morphologically altered macrophages,” but instead represent a distinct reprogrammed macrophage state. Compared with classically activated macrophages, they generally exhibit reduced phagocytic capacity but enhanced secretory activity and are thought to contribute more to local tissue organization and inflammatory niche formation than to direct, highly efficient phagocytic clearance. In a mycobacterial zebrafish model, Cronan et al. demonstrated that, during granuloma formation, macrophages can be induced to express epithelial‐associated molecules, such as E‐cadherin, and to form epithelial‐like junctional structures; disruption of macrophage E‐cadherin function led to disorganized granuloma architecture, increased immune‐cell entry into the lesion core, and reduced bacterial burden, suggesting that macrophage epithelialization may represent one mechanism underlying granuloma organization [181]. In addition to host‐derived signals, *M. tuberculosis* itself can actively shape this transformation process. Lin et al. found that the *M. tuberculosis* secreted antigen ESAT‐6 can bind to TLR2, activate iNOS/NO‐ and ROS‐related signaling, and reduce H3K27 trimethylation, thereby promoting the transition of macrophages into epithelioid cells. This finding indicates that the pathogen does not passively await granuloma formation but can directly reprogram macrophage differentiation trajectories [182].

LGCs are multinucleated giant cells formed by macrophage fusion and are important pathological hallmarks of tuberculosis and other granulomatous diseases. Early in vitro studies showed that human peripheral blood monocytes can form multinucleated giant cells when directly exposed to mycobacteria in the presence of soluble factors derived from T cells, and that this process depends on direct contact between mycobacteria and monocytes and is regulated by IFN‐γ [183]. Subsequently, Sakai et al., using a coculture system of human monocytes and autologous T cells, further demonstrated that LGC formation depends on close contact between monocytes and T cells, with CD4^+^ T cells exerting a stronger inductive effect than CD8^+^ T cells. Mechanistically, CD40–CD40L interactions and IFN‐γ are key driving factors, whereas the fusion‐related molecule DC‐STAMP is upregulated and directly participates in giant cell formation [184]. These findings suggest that the appearance of LGCs usually emerge after adaptive immunity has been established and large numbers of T cells have entered the lesion, and therefore they are better regarded as features of granuloma maturation and sustained tissue organization.

Overall, the formation of epithelioid cells and the generation of LGCs together mark the progression of tuberculous granulomas from “inflammatory cell aggregates” to “structured immune lesions.” The former primarily promotes lesion organization by enhancing cell adhesion and local barrier formation, whereas the latter reflects a deeper level of macrophage fusion and terminal differentiation under persistent antigenic stimulation and T‐cell help. In classical tuberculous granulomas, epithelioid cells are usually located in the lesion core or around the caseous necrotic area, forming a tightly arranged macrophage layer, whereas LGCs are scattered within these macrophage‐rich regions. Outside the zone, a lymphocytic cuff composed mainly of T cells and B cells gradually forms.

### 2.3 Fibrotic Containment and *M. tuberculosis* Persistence

In some granulomas, the local immune response does not progress toward efficient bacterial clearance, but instead shifts into a restrained state characterized by an immunosuppressive and tissue repair‐oriented microenvironment. This niche, shaped by suppressive myeloid cells, regulatory T cells, and TGF‐β‐dominated signaling, limits excessive inflammation but also reduces antimicrobial pressure, thereby promoting fibrotic containment and the persistent survival of *M. tuberculosis*.

Fibrotic containment is not merely a passive scar‐forming outcome, but an active structural program of lesion organization. In a minipig model of tuberculosis, Gil et al. showed that granuloma encapsulation is a key factor in limiting lesion progression and containing infection, providing direct evidence that fibrotic cuff formation contributes to the spatial restriction of mycobacterial spread [185]. Consistent with this concept, studies of tuberculous lesions have shown that TGF‐β signaling is closely associated with collagen deposition and tissue remodeling. Toossi et al. first reported increased TGF‐β production by monocytes from patients with active tuberculosis and demonstrated TGF‐β expression within pulmonary tuberculous granulomas [186]. Subsequently, Aung et al. showed that *M. tuberculosis* promotes bioactivation of latent TGF‐β1 at disease sites, indicating that this pathway is actively engaged in the granuloma microenvironment rather than being a passive byproduct of inflammation [187]. In nonhuman primates, DiFazio et al. further demonstrated spatially correlated distributions of active TGF‐β, pSMAD2/3, α‐SMA, and collagen deposition in granulomas, supporting a direct role for the TGF‐β/SMAD axis in the profibrotic remodeling of tuberculous lesions [188].

The execution of fibrotic remodeling likely involves multiple stromal and myeloid cell populations. Beyond classical fibroblast activation, systems biology analyses combined with experimental validation has suggested that macrophage‐to‐myofibroblast transition may contribute to the formation of the peripheral fibrotic cuff in granulomas, implying that macrophages themselves can be reprogrammed into collagen‐producing, myofibroblast‐like cells during lesion maturation [189]. In parallel, *M. tuberculosis* can directly stimulate profibrotic programs in lung stromal cells. Lee et al. showed that *M. tuberculosis* induces connective tissue growth factor expression in human lung fibroblasts through the TLR2–JNK–AP‐1 pathway, supporting the concept that bacilli actively engage fibroblast‐dependent tissue remodeling rather than being merely passively enclosed by host repair processes.

Although this fibrotic architecture helps restrain lesion expansion, it does not confer sterilizing immunity. Instead, it creates a dense and relatively inaccessible niche in which bacilli can persist under reduced immune and metabolic pressure. A major cellular correlate of this state is the foamy macrophage. Peyron et al. demonstrated, in human tuberculous granulomas and an in vitro human granuloma model, that foamy macrophages constitute a lipid‐rich reservoir in which *M. tuberculosis* adopts a dormant, nonreplicative phenotype [190]. Consistent with this concept, Kim et al. showed that granuloma caseation correlates with pathogen‐driven dysregulation of host lipid metabolism, indicating that altered lipid handling within the lesion microenvironment supports both pathological remodeling and bacillary persistence [191].

Persistence is further reinforced at the acellular level of the lesion. Caseum, the necrotic material that accumulates within closed nodules and fibrotic lesions, is increasingly recognized as a reservoir of drug‐tolerant persisting mycobacteria. Sarathy and colleagues showed that bacilli recovered from caseum exhibit profound tolerance to multiple antituberculosis drugs, indicating that lesion architecture itself can generate a pharmacologically protected persister niche [192]. Thus, fibrotic containment should be understood as a double‐edged process: although it spatially restricts bacillary dissemination and limits tissue‐destructive inflammation, it also contributes to the creation of a poorly perfused, poorly penetrable, immune‐constrained microenvironment that favors the long‐term survival of *M. tuberculosis*.

Taken together, fibrotic containment in tuberculosis reflects a state of incomplete but durable host control. Through TGF‐β‐driven repair signaling, collagen deposition, stromal and myeloid cell reprogramming, and the establishment of lipid‐rich and caseum‐based persister reservoirs, granulomas can simultaneously contain infection and preserve viable bacilli. This duality provides a pathological basis for long‐term *M. tuberculosis* persistence and helps explain why apparently stable lesions may later serve as the substrates for reactivation.

### 2.4 Breakdown of Granuloma Containment: Persister Reactivation, Liquefactive Necrosis, and Active Pulmonary Tuberculosis

Unlike fibrotic encapsulation and the relatively restricted state of persistence, some tuberculous granulomas progressively lose structural integrity and immunological stability during disease evolution, and instead enter a destructive trajectory characterized by aggravated caseous necrosis, liquefaction, cavity formation, and bronchogenic dissemination. At this stage, the lesion no longer primarily reflects “limited control,” but rather represents a breakdown of containment. During this process, persisting bacilli confined within the lesion regain metabolic activity, followed by sustained inflammatory amplification, structural tissue destruction, and softening of the lesion core, ultimately driving the development and transmission of active pulmonary tuberculosis (Figure 1).

Persister reactivation is one of the key early events following the breakdown of containment. The rabbit latent tuberculosis model provides direct evidence for this process: after aerosol infection, some animal maintain a low bacterial burden and relatively stable lesions for prolonged periods, but following glucocorticoid‐induced immunosuppression, culture positivity and disease relapse re‐emerge, indicating that *M. tuberculosis* within restricted lesions is not completely eradicated but instead resumes replication when host control is weakened [193]. Consistent with this, prospective cohort studies have shown that, before adolescents with latent tuberculosis progress to active disease, peripheral blood already displays a Type I IFN‐related transcriptional signature driven by neutrophil‐associated inflammatory programs, suggesting that reactivation is not merely a local pathological event confined to the lesion, but is accompanied by systemic immune disequilibrium [194, 195]. From the bacterial perspective, resuscitation‐promoting factors, a family of cell wall‐active enzymes, are thought to participate in the transition of *M. tuberculosis* from dormancy to active growth, and thus constitute an important molecular basis for persister reactivation [196]. Nevertheless, other bacterial factors that directly drive reactivation remain incompletely defined.

After persisting bacilli resume replication, inflammatory amplification and tissue‐destructive programs within the lesion microenvironment are further intensified. Among these processes, sustained disruption of the extracellular matrix barrier is one of the central pathological bases of containment failure. Elkington and colleagues showed that MMP‐1 is a major effector driving collagen degradation and lung tissue destruction in human pulmonary tuberculosis; in transgenic mice expressing human MMP‐1, *M. tuberculosis* infection induces more pronounced alveolar structural damage and collagen breakdown [64]. These findings indicate that the transition of granulomas from “closed, restrictive lesions” to “destructive, open lesions” is not merely a passive consequence of increasing bacterial burden but is accompanied by host‐driven proteolytic and matrix‐degrading programs. In this context, neutrophil‐rich inflammation further amplifies tissue injury. Ong and colleagues found that the hypoxic environment within tuberculous lesions markedly enhances neutrophil‐mediated degradation of Type I collagen, gelatin, and elastin, together with increased secretion of MMP‐8, MMP‐9, and neutrophil elastase [197]. Subsequent work showed that MMP‐8 is predominantly derived from neutrophils and is associated with matrix destruction and disease severity in respiratory samples, indicating that neutrophils are not mere bystanders but important executors of tissue damage preceding cavity formation [198].

In addition to proteolysis, lipid disequilibrium and the continued evolution of caseous necrosis also provide an important basis for liquefaction. As infection progresses, macrophages within the lesion gradually undergo lipid reprogramming and become foamy macrophages, characterized by marked accumulation of cholesterol esters, neutral lipids, and other lipid species. Studies of human tuberculous granulomas have shown that caseating lesions are associated with markedly increased host lipid metabolism, and that necrotic cores are enriched in cholesterol, cholesterol esters, triglycerides, and related lipid components [191]. Such a lipid‐rich, hypoxic microenvironment with impaired clearance not only provides nutrients and shelter for the persistent survival of *M. tuberculosis*, but also gradually shifts the necrotic core from a solid caseous state toward a more prone to softening and liquefaction.

Overall, liquefactive necrosis of granuloma is not simply the natural extension of a single necrotic process, but rather the integrated consequence result of persister reactivation, inflammatory disequilibrium, matrix degradation, neutrophil‐amplified tissue injury, and the continued evolution of lipid‐rich necrosis. This process marks the breakdown of the host restrictive control over the lesion and ultimately transforms a relatively closed tuberculous granuloma into an active pulmonary tuberculosis lesion characterized by high bacillary burden and high transmission potential.

## 3 Host–Pathogen Immune Interplay: Mechanistic Integration

**FIGURE 4:**
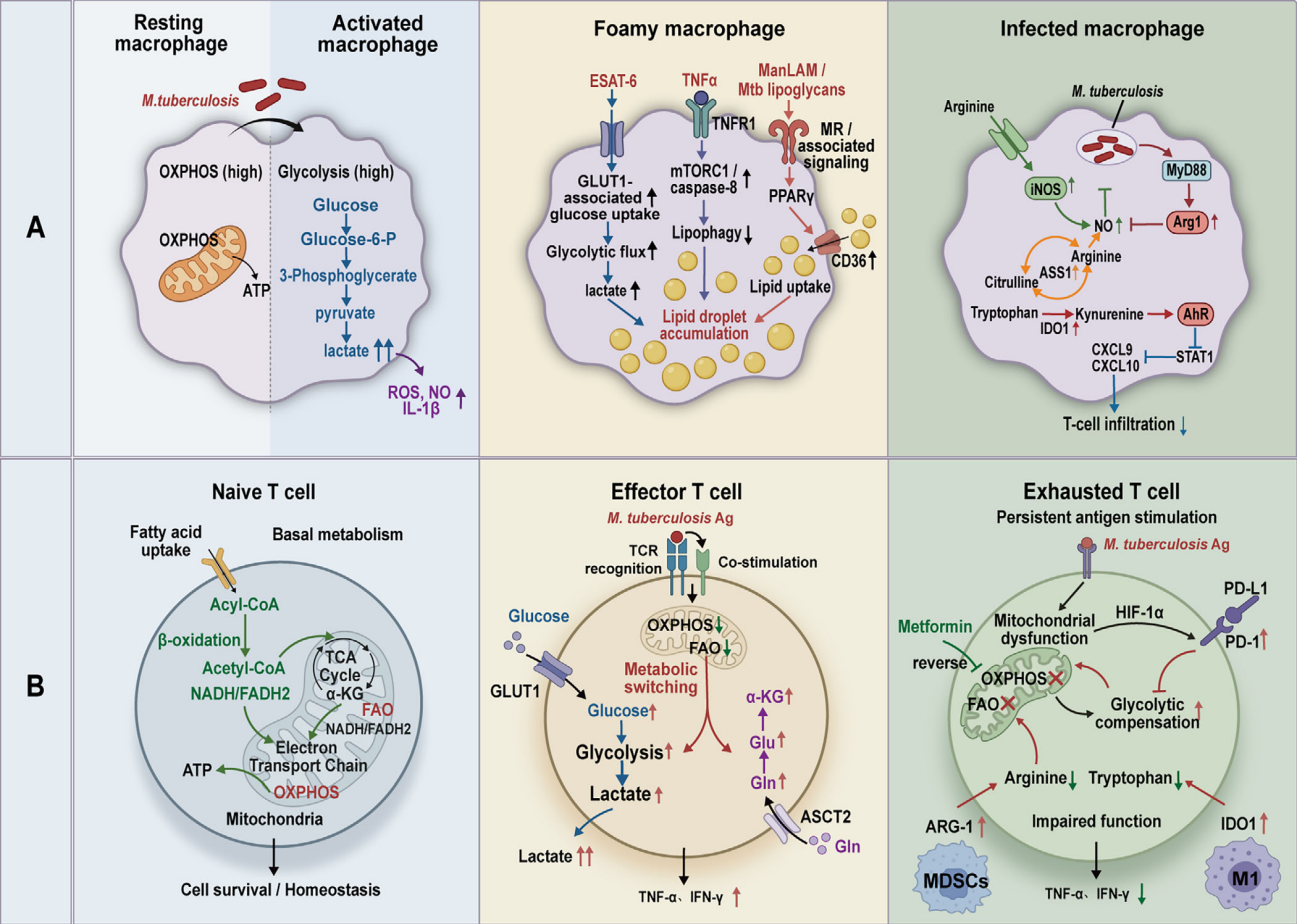
Metabolic reprogramming of macrophages and T cells within tuberculous granulomas. (A) Macrophage metabolic reprogramming. (1) In resting macrophages, energy production largely depends on oxidative phosphorylation (OXPHOS). Upon *M. tuberculosis* infection, macrophages shift toward glycolysis, and enhanced generation of antimicrobial mediators such as ROS, NO, and IL‐1β; (2) *M. tuberculosis* also promotes foamy macrophage formation by promoting lipid accumulation: ESAT‐6 enhances glucose transporter 1 (GLUT1)‐associated glucose uptake and glycolytic flux, thereby increasing metabolic substrates for lipid biosynthesis; TNF‐α–TNFR1 signaling activates the mTORC1/caspase‐8 pathway, suppresses lipophagy; and ManLAM and mycobacterial lipoglycans activate the mannose receptor (MR)–PPARγ–CD36 axis to further enhance lipid uptake and storage; (3) *M. tuberculosis* also reshapes amino acid metabolism by regulating arginine and tryptophan pathways. Arginine uptake and argininosuccinate synthase 1‐mediated citrulline recycling sustain NO generation, whereas MyD88‐dependent induction of Arg1 competes with iNOS for arginine and limits antimicrobial activity. In parallel, indoleamine 2,3‐dioxygenase 1 (IDO1)‐mediated tryptophan catabolism generates kynurenine, which activates aryl hydrocarbon receptor (AhR) signaling and suppresses the STAT1–CXCL9/CXCL10 axis, thereby reducing T‐cell infiltration. (B) T cell metabolic reprogramming. (1) Naïve T cells largely depends on oxidative phosphorylation (OXPHOS) and fatty acid oxidation (FAO); (2) Upon recognition of *M. tuberculosis* antigens, activated T cells undergo metabolic switching characterized by increased glucose uptake through GLUT1, enhanced aerobic glycolysis, lactate production, and glutamine(Gln) utilization through ASCT2. These metabolic changes support clonal expansion and the production of effector cytokines; (3) Persistent antigen stimulation and the metabolically granuloma microenvironment drive progressive T‐cell dysfunction. Exhausted T cells exhibit impaired mitochondrial OXPHOS and FAO, compensatory glycolytic activity. The PD‐1/PD‐L1 axis further suppresses T‐cell metabolic fitness and effector function. In addition, myeloid‐derived suppressor cells (MDSCs) and IDO1^+^ macrophages drive local arginine deprivation through arginase1 (ARG1) and tryptophan catabolism, respectively, further impairing T cell function.

During tuberculosis, immune cells exhibit marked plasticity and are dynamically reshaped by persistent *M. tuberculosis* stimulation and the complex granuloma microenvironment. This plasticity determines whether immune cells maintain protective antimicrobial functions or are redirected toward dysfunctional, suppressive, or tissue‐damaging states. Mechanistically, this plasticity is governed by interconnected regulatory networks, including metabolic reprogramming (Figure 4), epigenetic remodeling, and host–cell death pathways.

### 3.1 Metabolic Reprogramming

#### 3.1.1 Macrophage Metabolic Reprogramming

Under resting conditions, macrophages primarily depend on oxidative phosphorylation (OXPHOS) for energy production. However, upon *M. tuberculosis* stimulation, their metabolic program shifts toward glycolysis [199] (Figure 4A). This metabolic rewiring represents a key component of the rapid innate immune response, supporting the production of ROS and NO, and promoting the activation of proinflammatory pathways such as IL‐1β signaling, thereby enhancing the ability of macrophages to restrict intracellular pathogens.

*M. tuberculosis* can further exploit macrophage metabolic plasticity by redirecting lipid metabolism toward a lipid‐laden state that favors foamy macrophage formation (Figure 4A). Mechanistically, *M. tuberculosis* promotes foamy macrophage formation through coordinated remodeling of glucose and lipid metabolism. The virulence factor ESAT‐6 enhances GLUT1‐mediated glucose uptake and perturbs glycolytic flux, thereby providing metabolic substrates that may favor lipid biosynthesis and foam‐cell differentiation [200]. In parallel, *M. tuberculosis* reshapes macrophage lipid homeostasis by increasing lipid uptake and storage while limiting lipid mobilization. TNF receptor‐associated signaling can activate mTORC1 and caspase‐8, thereby suppressing lipophagy and enhancing lipid droplet accumulation [201]. In addition, ManLAM engages pattern‐recognition receptor‐associated pathways and is linked to activation of the PPARγ–CD36 lipid uptake program, further promoting lipid storage [202]. Moreover, UreC(Rv1850) activates the IFN‐β pathway and upregulates the scavenger receptor scavenger receptor‐A1, increasing lipid uptake and lipid droplet formation [203]. Through these convergent mechanisms, *M. tuberculosis* drives macrophages toward a lipid‐laden foamy phenotype, creating a metabolically permissive intracellular niche that may support bacterial persistence and granuloma progression. Following the necrosis of foamy macrophages, large amounts of cellular debris, uncleared lipids, and mineral components are released and gradually accumulate in the lesion center, thereby promoting the development of a caseous necrotic core characterized by high lipid content, acidity, and hypoxia. Collectively, macrophage lipid metabolic reprogramming tightly links intracellular metabolic alterations to the histopathological evolution of granulomas and constitutes a key mechanism underlying persistent tuberculosis infection.

Beyond glycolytic and lipid metabolic remodeling, *M. tuberculosis* infection also reshapes amino acid metabolism (Figure 4A). Arginine metabolism represents a central immunometabolic checkpoint. During mycobacterial infection, macrophages import extracellular arginine to support iNOS‐dependent NO production, and argininosuccinate synthase 1‐mediated citrulline recycling is required to sustain NO generation and control intracellular mycobacterial infection under arginine‐limited conditions [204]. In parallel, *M. tuberculosis* can induce Arg1 expression in infected macrophages through MyD88‐dependent autocrine–paracrine cytokine signaling, thereby influencing arginine utilization and macrophage antimicrobial programs [205]. In tuberculosis granulomas, Arg1‐expressing macrophages have been shown to spatially regulate inflammation, bacterial growth, and tissue pathology, suggesting that arginine metabolism balances bactericidal activity with immunoregulatory and tissue‐protective functions [206]. Tryptophan metabolism through the IDO1–kynurenine axis further contributes to immune regulation during tuberculosis. *M. tuberculosis* infection induces IDO1 expression and activation of the tryptophan–kynurenine pathway [207]. More recently, kynurenine–AhR signaling was shown to suppress the STAT1–CXCL9/CXCL10 axis, thereby reducing T‐cell infiltration and delaying protective T‐cell responses during tuberculosis [153].

In parallel, *M. tuberculosis* infection profoundly alters metal metabolism. Host macrophages deploy nutritional immunity by limiting bacterial access to essential metals such as iron [208], while also using zinc and copper intoxication within phagosomal compartments as antimicrobial strategies [209, 210]. Conversely, *M. tuberculosis* has evolved sophisticated metal acquisition, detoxification, sequestration, and efflux systems to withstand metal deprivation or toxicity and maintain intracellular survival [209, 211].

Together, these metabolic programs do not act in isolation but form an integrated immunometabolic network that determines whether infected macrophages maintain a bactericidal phenotype or are redirected toward a permissive, dysfunctional, immunosuppressive, or tissue‐damaging state.

#### 3.1.2 Metabolic Reprogramming of T Cells

T cells are central effector cells in antituberculosis immunity, and their activation, expansion, and maintenance of effector functions depend on dynamic metabolic regulation (Figure 4B). During the early phase of activation, naïve T cells rapidly switch from a quiescent metabolic program primarily supported by OXPHOS and fatty acid oxidation to an activated state characterized by enhanced aerobic glycolysis, thereby supporting clonal expansion and the production of effector molecules [212]. In addition to glucose metabolism, accumulating evidence suggests that glutamine metabolism has emerged as an important contributor for T‐cell responses against mycobacterial infection [213]. These findings indicate that protective T‐cell immunity in tuberculosis depends not only on glucose metabolism but also on amino acid metabolism. However, once T cells migrate into the granulomatous microenvironment, their metabolic state undergoes further remodeling. Tuberculous lesions are characterized by limited nutrient availability, restricted glucose access, hypoxia, lactate accumulation, and local acidification, all of which together create a metabolically unfavorable environment for effector T cells. Consistent with this concept, Whittington et al. demonstrated that the acidic microenvironment in tuberculosis markedly suppresses the production of key antimicrobial cytokines, including TNF‐α and IFN‐γ, while enhancing programs associated with matrix degradation and tissue destruction [214]. Collectively, these observations suggest that, in tuberculosis, T cells do not simply remain functional once activated; rather, they progressively encounter increasing difficulty in sustaining both high glycolytic activity and a robust effector state within lesions.

As infection progresses into the chronic stage, accumulating evidence indicates that T cells gradually fail to maintain metabolic adaptation, ultimately entering a functionally exhausted state. Studies have shown that, during disease progression, mitochondrial function in CD8^+^ T cells becomes progressively impaired, as evidenced by reduced oxidative metabolic capacity and increased dependence on glycolysis [215]. Using genetic models, Wu et al. further demonstrated that mitochondrial insufficiency can drive the transition of progenitor exhausted T cells toward terminally exhausted T cells through an HIF‐1α‐dependent pathway [216].

Although enhanced glycolysis may transiently support the production of inflammatory cytokines, sustained metabolic imbalance ultimately leads to insufficient bioenergetic reserves, progressive mitochondrial dysfunction, and increased expression of inhibitory receptors. Notably, metformin has been shown to reverse the bioenergetic decline of T cells induced by *M. tuberculosis*, thereby generating a population of *M. tuberculosis*‐specific CD8^+^ T cells with improved metabolic fitness [215]. This finding suggests that exhaustion‐associated metabolic abnormalities in T cells retain a degree of plasticity and may therefore be amenable to pharmacological intervention. Moreover, persistent activation of the PD‐1/PD‐L1 axis can further suppress glycolysis and amino acid metabolism in T cells, thereby impairing effector differentiation and functional maintenance [217]. Collectively, these findings support the view that metabolic dysregulation is not merely a downstream consequence of established exhaustion; rather, it may precede overt functional impairment and may represent an early hallmark as well as an important driver of T‐cell exhaustion.

In addition to cell‐intrinsic metabolic defects, T‐cell dysfunction in tuberculosis is further shaped by myeloid cell‐driven remodeling of amino acid metabolism. Studies have shown that Arg1^+^ MDSCs in tuberculous lesions competitively deplete arginine from the local microenvironment, leading to reduced expression of the CD3ζ chain and impaired TCR signaling, thereby suppressing T‐cell effector function [218, 219, 220]. Beyond disrupting receptor signaling, arginine deprivation mediated by Arg1^+^ MDSCs may further impair mTOR‐dependent mitochondrial metabolic programs, thus promoting metabolic maladaptation in T cells and driving their progression toward an exhausted state [221]. Meanwhile, *M. tuberculosis* infection and associated inflammatory cues can induce macrophages and other myeloid cells to upregulate IDO1, thereby redirecting local tryptophan metabolism into the kynurenine pathway. Notably, studies have demonstrated that tryptophan depletion can directly inhibit T‐cell proliferation and attenuate immune responses [207, 222, 223]. Collectively, these findings indicate that myeloid cell‐driven reprogramming of arginine and tryptophan metabolism constitutes an important mechanism through which the tuberculous microenvironment promotes T‐cell dysfunction.

### 3.2 Epigenetic and Epigenetic‐Related Posttranslational Reprogramming

Epigenetic reprogramming has emerged as an important mechanism by which *M. tuberculosis* modulates the plasticity of host immune cells. Through secreted effector molecules and the host signaling pathways they engage, *M. tuberculosis* can remodel the epigenetic landscape of macrophages and T cells, thereby suppressing antimicrobial activity and antigen presentation and ultimately promoting cellular states that favor long‐term intracellular persistence.

During the innate immune phase, epigenetic reprogramming contributes to *M. tuberculosis*‐mediated impairment of phagocyte uptake and bactericidal activity. For example, the *M. tuberculosis*‐secreted N‐acetyltransferase Eis mediates N‐acetylation of DUSP16/MKP‐7 at Lys55, thereby inhibiting phagosome maturation in infected macrophages [224]. In addition, *M. tuberculosis* protein PRT enhance the activity of the host methyltransferase G9a, resulting in increased H3K9me2/3 levels and transcriptional silencing of autophagy‐related genes. Consistent with this, treatment with the G9a inhibitor UNC0638 reduces H3K9me2/3 levels, restores the expression of autophagy‐associated genes, and consequently enhances autophagic clearance [48].

At the adaptive immune level, *M. tuberculosis* can further impair effective T‐cell priming by epigenetically suppressing antigen presentation. *M. tuberculosis* and its 19‐kDa lipoprotein induce deacetylation of histones H3 and H4 at the CIITA promoter through a TLR2‐dependent mechanism, thereby directly repressing CIITA transcription and subsequently downregulating MHC‐II expression [225, 226]. In addition, *M. bovis* infection has been reported to promote NO‐dependent binding of KLF4 to the CIITA promoter, accompanied by recruitment of the histone methyltransferase EZH2, which enhances H3K27me3 deposition at this region and further suppresses CIITA transcription and MHC‐II expression [227]. Collectively, these findings indicate that *M. tuberculosis* undermines antigen presentation through epigenetic mechanisms involving histone deacetylation and methylation, thereby limiting the initiation of effective adaptive immune responses.

## 4 Implications for Vaccine Design and Adjunctive Therapies Targeting Host–Pathogen Interactions

Although *M. tuberculosis* infection elicits intricate innate and adaptive immune responses, the pathogen has evolved multifaceted immune evasion strategies to ensure its persistence and long‐term survival (Table 1). These mechanisms include subverting phagosomal maturation and intracellular killing, delaying, and attenuating T cell priming, and exploiting the granuloma to establish an immunosuppressive and metabolically restricted microenvironment. Therefore, elucidating the dynamic interplay between host immunity and *M. tuberculosis* is pivotal for deciphering the immunological underpinnings of tuberculosis progression and for informing the design of novel vaccines and adjunctive therapies.

**TABLE 1:** Major immune evasion mechanisms of *M. tuberculosis*.

Immune evasion category	*M. tuberculosis* components	Host target or affected cellular process	Mechanism of immune evasion	References
Arrest of phagosome maturation	ManLAM	PI3K/Rab5 signaling	Inhibits Ca^2^^+^/calmodulin signaling and Rab5‐dependent PI3K recruitment, thereby blocking de novo PI3P synthesis and arresting phagosome maturation	[29]
	SapM	PI3P	Hydrolyzes phagosomal PI3P, thereby disrupting PI3P‐dependent phagosome maturation	[30]
	PtpA	V‐ATPase	Inhibits V‐ATPase recruitment and proton pump activity, leading to impaired phagosomal acidification	[31]
	TDM	Host phagosomal pH regulatory	Interferes with host pH‐regulatory mechanisms and delays phagosomal acidification	[32]
	PIMs	PI3K/EEA1‐dependent lysosomal trafficking	Disrupts delivery of lysosomal component to phagosomes	[33]
	PknG	RILP–HOPS complex	Inhibits recruitment of the HOPS complex, thereby preventing lysosomal docking and phagolysosome formation	[34]
Resistance to oxidative and nitrosative stress	KatG	Peroxides	Detoxifies hydrogen peroxide and peroxide‐derived oxidative stress	[37]
	SodA/SodC	Superoxide	Scavenges superoxide radicals generated during the host oxidative burst	[38, 39]
	AhpC/AhpD	Peroxides/peroxynitrite	Detoxifies peroxides and peroxynitrite	[40, 41]
	Thioredoxin (Trx);mycothiol (MSH)/WhiB3	Intrabacterial redox homeostasis	Maintains redox balance under immune pressure	[42, 43, 44]
Suppression of autophagy	ManLAM	VPS34/PI3P axis; Ca^2^^+^–mTOR/ULK1; beclin‐1 pathways	Impairs autophagy initiation through multiple signaling axes	[45]
	PknG	Rab14‐dependent autophagosome maturation pathway	Blocks autophagosome maturation into autolysosomes and disrupts autophagic flux	[46]
	SapM	Raptor–mTORC1	Inhibits autophagy initiation through a raptor–mTORC1‐dependent mechanism	[47]
	PRT	Atg5/Atg7	Suppresses transcription of autophagy‐related genes via epigenetic remodeling	[48]
Modulation of apoptosis	ManLAM	Ca^2^^+^‐dependent signaling; caspase cascade; Bcl‐2‐associated survival signaling	Inhibits mitochondria‐related apoptotic signaling by dampening proapoptotic pathways	[50]
	PtpA	Caspase‐3	Restricts caspase‐3 activation and suppresses apoptosis	[51]
	PE6 (Rv0335c)	Bax/Bak pathway apoptotic pathway	Induces a Bax/Bak‐associated mitochondrial apoptotic pathway	[52]
Suppression of pyroptosis	PknF	NLRP3 inflammasome	Inhibits inflammasome activation and reduces pyroptotic signaling	[53]
	PtpB	GSDMD‐N/plasma membrane phospholipids	Alters membrane phospholipids and blocks GSDMD‐N pore formation	[54]
	ESX‐1 secretion system	AIM2 inflammasome	Suppresses AIM2 inflammasome‐mediated pyroptosis	[55]
Epithelial adhesion and entry	HBHA	Epithelial heparan sulfate proteoglycans	Promotes bacterial attachment to epithelial cells	[61, 62, 63, 64]
Neutrophil oxidative killing resistance	H_2_S; EGT	HOCl	Directly neutralizes hypochlorous acid and limit oxidative damage	[86, 87]
Impairment of DC maturation and T‐cell priming	ManLAM; Di‐O‐acyl trehalose	DC‐SIGN; DCIR; Dectin‐2; Mincle	Skews DCs toward a tolerogenic phenotype with reduced costimulatory molecules and MHC II, and increased IL‐10 and IDO	[96, 97, 98, 99]
	ESAT‐6; Hip1; Zmp1; PE‐PGRS47; Acr‐1	DC maturation/antigen presentation machinery	Disrupts maturation and antigen presentation capacity of DCs	[100, 101, 102, 103, 104]
Delay of T‐cell priming	EsxH	ESCRT machinery in antigen‐presenting cells	Disrupts ESCRT function, impairs antigen processing/MHC II presentation, delaying effective CD4^+^ T‐cell priming	[150]
	ESAT‐6	β2‐microglobulin; MHC I heavy chain assembly	Binds β2M and prevents proper MHC I assembly, causing ER retention and impairing CD8^+^ T‐cell activation	[151]
	Hip1	GroEL2/DC maturation pathway	Cleaves GroEL2 into a poorly immunostimulatory form, compromising DC maturation and antigen presentation	[102]
	PE_PGRS47	Host autophagy pathway/MHC Class II presentation	Suppresses host autophagy, thereby weakening MHC Class II‐restricted antigen presentation and limiting CD4^+^ T‐cell priming	[101]
	PDIM	DC costimulatory and cytokine responses	Inhibits CD86 and IL‐12p40 expression; weakening costimulatory signaling and Th1‐polarizing cytokine production	[152]
Promotion of Treg‐mediated suppression	Rv1272c	Treg cells/lipid metabolic pathway	Upregulated under hypoxia, promotes lipid uptake and linoleic acid release, enhancing Treg function and delaying effector T‐cell accumulation	[155]
Impairment of T‐cell effector function	Rv0884c (SerC)	WDR24–mTORC1–T‐bet axis	Promotes D‐serine production under hypoxia, directly suppressing the WDR24–mTORC1–T‐bet pathway in CD8^+^ T cells and reducing IFN‐γ production	[156]
Interference with B‐cell function	LAM	TLR2/4‐MyD88 signaling pathway	Drives expansion of IL‐10‐producing regulatory B cells, suppressing Th1 polarization and favoring Th2‐biased responses	[175]
	PtpA	SYK; BTK	Dephosphorylates key BCR‐signaling kinases, impairing B‐cell activation, plasma‐cell differentiation, and antibody secretion	[176]
	ESAT‐6	TLR2/iNOS‐NO and ROS signaling / H3K27me3	Binds TLR2, activates iNOS/NO‐ and ROS‐related signaling, and reduces H3K27 trimethylation, thereby promoting macrophage transition into epithelioid cells and granuloma organization	[182]

Abbreviations: Acr‐1, alpha‐crystallin 1; AhpC/AhpD, alkyl hydroperoxide reductase C/D; AIM2, absent in melanoma 2; Atg5/Atg7, autophagy‐related protein 5/7; Bcl‐2, B‐cell lymphoma 2; BCR, B‐cell receptor; BTK, Bruton's tyrosine kinase; DC, dendritic cell; DCIR, dendritic cell immunoreceptor; DC‐SIGN, dendritic cell‐specific intercellular adhesion molecule‐3‐grabbing nonintegrin; EEA1, early endosome antigen 1; EGT, ergothioneine; ER, endoplasmic reticulum; ESAT‐6, early secreted antigenic target of 6 kDa; ESCRT, endosomal sorting complex required for transport; ESX‐1, ESAT‐6 secretion system 1; GSDMD‐N, N‐terminal gasdermin D; H2S, hydrogen sulfide; HBHA, heparin‐binding hemagglutinin adhesin; HOCl, hypochlorous acid; HOPS, homotypic fusion and protein sorting; IDO, indoleamine 2,3‐dioxygenase; IFN‐γ, interferon‐γ; IL, interleukin; iNOS, inducible nitric oxide synthase; KatG, catalase‐peroxidase; LAM, lipoarabinomannan; ManLAM, mannose‐capped lipoarabinomannan; MHC, major histocompatibility complex; MSH, mycothiol; mTOR, mechanistic target of rapamycin; MyD88, myeloid differentiation primary response 88; NLRP3, NOD‐like receptor family pyrin domain‐containing 3; NO, nitric oxide; PDIM, phthiocerol dimycocerosate; PI3K, phosphoinositide 3‐kinase; PI3P, phosphatidylinositol 3‐phosphate; PIMs, phosphatidylinositol mannosides; PknF, protein kinase F; PknG, protein kinase G; PRT, phosphoribosyltransferase; PtpA, protein tyrosine phosphatase A; PtpB, protein tyrosine phosphatase B; RILP, Rab‐interacting lysosomal protein; RNS, reactive nitrogen species; ROS, reactive oxygen species; SapM, secreted acid phosphatase M; SodA/SodC, superoxide dismutase A/C; SYK, spleen tyrosine kinase; TDM, trehalose dimycolate; TLR, Toll‐like receptor; Treg, regulatory T cell; Trx, thioredoxin; ULK1, Unc‐51‐like autophagy activating kinase 1; V‐ATPase, vacuolar‐type H^+^‐ATPase; VPS34, vacuolar protein sorting 34; WDR24, WD repeat domain 24; WhiB3, white B‐like protein 3; Zmp1, zinc metalloprotease 1; β2M, β2‐microglobulin.

### 4.1 Vaccine Design Based on Trained Immunity

Given the limited efficacy of the BCG vaccine in preventing adult pulmonary tuberculosis, the development of more efficacious alternatives remains an urgent clinical priority. However, tuberculosis vaccine research is impeded by substantial challenges, notably the absence of defined correlates of protection (CoPs), which remains a major barrier to vaccine development. Historically, most next‐generation vaccine strategies have predominantly focused on amplifying antigen‐specific adaptive immunity—for instance, by promoting naïve T cell activation or driving the expansion of memory T cell pools [228]. Nevertheless, accumulating evidence suggests that augmenting adaptive immunity alone is insufficient to confer sterilizing immunity against *M. tuberculosis*. For example, despite its robust immunogenicity, the candidate vaccine MVA85A, which was designed to boost IFN‐γ responses, failed to provide significant additional protection in clinical trials [229]. Driven by a deeper mechanistic understanding of host–pathogen interactions, tuberculosis vaccine design is increasingly moving beyond the goal of simply amplifying antigen‐specific adaptive immunity toward the induction of coordinated innate and adaptive protective responses.

Trained immunity, also termed innate immune memory, refers to the long‐term functional reprogramming of innate immune cells triggered by exogenous or endogenous stimuli. Following this initial priming, these cells exhibit an altered, heightened responsiveness upon subsequent exposure to homologous or heterologous challenges. This hyper‐responsiveness is characterized by the increased production of proinflammatory cytokines, such as IL‐1β, IL‐6, and TNF‐α, conferring enhanced cross‐protection against secondary infections [230, 231, 232, 233]. Monocytes isolated from BCG‐vaccinated healthy volunteers show enhanced inflammatory cytokine production upon ex vivo stimulation with heterologous bacterial and fungal pathogens [234]. Importantly, this nonspecific protective effect is sustained for at least 1 year postvaccination, driven primarily by mechanisms of glycolytic reprogramming and epigenetic rewiring [235, 236, 237]. Given the important role of innate immune cells in phagocytosing and killing *M. tuberculosis* as well as in initiating T‐cell‐mediated adaptive immune responses, the development of next‐generation tuberculosis vaccines should not be limited to enhancing antigen‐specific adaptive immunity, but should also fully consider and harness trained immunity as an important protective mechanism.

The potential contribution of trained immunity is also supported by the protective efficacy observed in several late‐stage clinical vaccine candidates. A prominent example is the recombinant subunit vaccine M72/AS01E, currently undergoing Phase III clinical evaluation. This vaccine comprises the recombinant fusion protein M72, derived from *M. tuberculosis* antigens Mtb32A (Rv0125/PepA) and Mtb39A (Rv1196/PPE18), formulated with the liposomal adjuvant AS01E containing monophosphoryl lipid A (MPL) and QS‐21 [238]. Results from its Phase IIb clinical trial demonstrated a significant reduction in the incidence of active pulmonary tuberculosis in the vaccinated cohort compared with the placebo group, achieving an overall vaccine efficacy of 54% (95% CI: 2.9–78.2) [239]. Subsequent mechanistic investigations revealed that immunization with the AS01E‐adjuvanted vaccine induces epigenetic modifications in monocyte and DC subsets [240]. These findings raise the possibility that AS01E‐induced innate immune reprogramming may contribute to the protective efficacy of M72/AS01E, although the precise molecular mechanisms require further investigation.

Similarly, data from Phase II clinical trials of MTBVAC and VPM1002 further suggest that remodeling innate immunity may be an important component of rational tuberculosis vaccine design. By preserving a more comprehensive repertoire of antigens and pathogen‐associated molecular patterns, MTBVAC not only exhibits enhanced immunogenicity but also induces trained immunity in monocytes via metabolic and epigenetic reprogramming, thereby augmenting innate defense mechanisms against subsequent stimuli [241, 242]. In parallel, VPM1002 promotes robust intracellular innate recognition and subsequent T‐cell responses by enhancing phagosomal acidification, cytosolic antigen release, and antigen cross‐presentation. These properties may contribute to its improved immunogenicity and favorable safety profile relative to conventional BCG in clinical evaluation [243]. Collectively, these findings suggest that the optimization of next‐generation tuberculosis vaccines should incorporate strategies that modulate innate immune networks to establish durable protective immunity.

### 4.2 Therapeutic Strategies: Targeting Immune Evasion and Dysregulation

Elucidating the molecular mechanisms by which *M. tuberculosis* evades and reshapes host immunity provides a rationale for the development of adjunctive therapeutic strategies. When combined with direct‐acting antimicrobial agents, these approaches may shorten treatment duration, improve therapeutic outcomes, and reduce persistent *M. tuberculosis* reservoirs. This section summarizes representative strategies aimed at correcting *M. tuberculosis*‐induced dysfunction of host immune cells and restoring the balance between protective and pathological immune responses (Table 2).

**TABLE 2:** Tuberculosis vaccine candidates and therapeutic agents discussed in this review.

Candidate/agent	Target	Mechanism of action	Developmental stage	References
M72/AS01E	/	Induces significant epigenetic remodeling in monocytes and dendritic cells	Phase IIb trial (completed)	[238, 239]
MTBVAC	/	Induces trained immunity in monocytes through metabolic and epigenetic reprogramming	Phase III trial (ongoing)	[241, 242]
VPM1002	/	Enhances phagosomal acidification, cytosolic antigen release, and antigen cross‐presentation	Phase III trial (ongoing)	[243]
Tetrahydrobenzothiophene derivatives	PknG	Inhibit PknG activity, restore phagosome–lysosome fusion	In vitro study	[244]
(2E)‐1‐(2′‐hydroxyphenyl)‐3‐(1‐naphthyl)‐2‐propen‐1‐one	PtpA	Block PtpA activity, relieve interference with VPS33B‐mediated endolysosomal trafficking	In vitro study	[245]
L‐ascorbic acid, 2‐phospho‐L‐ascorbic acid, tyrphostin 51, galloflavin, YM‐26734	SapM	Impairs SapM activity to relieve its inhibition of phagosome maturation	In vitro study	[246, 247]
ZXL1	ManLAM	Relieve ManLAM‐mediated inhibition of dendritic cell maturation and upregulate MHC‐II, CD40	In vitro study	[248]
Rapamycin, everolimus	mTORC1	Inhibit mTORC1, promote autophagosome formation, and enhance macrophage bactericidal activity	In vivo study	[249, 250]
Metformin, AICAR	AMPK–mTORC1/autophagy	Activate AMPK, indirectly suppress mTORC1, and enhance autophagy‐related gene expression	In vivo study	[251, 252]
Simvastatin and other statins	AMPK–mTORC1–TFEB axis	Induce autophagy and promote intracellular bacterial clearance	In *viv* study	[253]
1,25‐(OH)2D_3_	Vitamin D receptor/autophagy	Induce cathelicidin expression and activate beclin‐1, Atg5, and autophagy pathways	In vivo study	[254]
Baicalin; epigallocatechin‐3‐gallate; honokiol	Autophagy pathway	Activate autophagy‐related signaling networks	In vivo study	[255, 256, 257]
Diethyldithiocarbamate; pyrrolidine dithiocarbamate	SODs	Chelate metals and interfere with metal‐dependent antioxidant defense	In vitro study	[258]
Testosterone 3(E),17‐dioxime	AhpD	Inhibits AhpD‐mediated electron transfer to AhpC, weakening peroxide detoxification	In vitro study	[259]
Auranofin, Ebselen, PX‐12	TrxB2	Disrupt thiol‐redox homeostasis and impair bacterial redox balance	In vivo study	[260, 261, 262]
AC2P36	Thiol metabolism	Depletes bacterial thiols, kills *M. tuberculosis* under acidic conditions, and potentiates standard drugs	In vitro study	[263]
Vitamin C	Redox stress/iron‐dependent ROS generation	Promotes Fenton chemistry and increases oxidative damage in *M. tuberculosis*	In vitro study	[264]
Clofazimine	Redox cycling	Generates ROS and enhances antimycobacterial activity	Approved; clinical re‐evaluation	[265]
IFN‐γ, GM‐CSF	Host macrophage oxidative response	Enhance ROS/RNS release by promoting macrophage activation	Clinical evaluation	[266, 267, 268]
Navitoclax	Host Bcl‐2‐mediated apoptosis pathway	Promotes protective apoptosis and improves bacterial clearance	In vivo study	[269]
Cyclosporin A; miR‐1281	Cyclophilin D (CypD)	Suppress CypD‐mediated mitochondrial permeability transition,	In vitro study	[270]
1‐Methyl‐D‐tryptophan; COX‐2 inhibitors	IDO/COX‐2	Alleviate MDSC‐mediated immunosuppression and improve T‐cell function	In vivo study	[271, 272]
Roflumilast	Phosphodiesterase 4 (PDE4)	Modulates MDSCs function and enhances isoniazid efficacy	In vivo study	[273]
JHU083	Glutamine metabolism	Restrict host glutamine supply to *M. tuberculosis*, reduce immunosuppressive myeloid cells, increase effector T cells	In vivo study	[274]
IL‐7	T‐cell expansion	Promote CD4^+^ T‐cell proliferation and expands naïve/central memory T‐cell	In vivo study	[275]
IL‐15	T‐cell activation	Enhances CD4^+^/CD8^+^ T‐cell responses, and strengthens Th1 immunity	In vivo study	[276]
IL‐12	Th1 differentiation	Promote Th1 differentiation and IFN‐γ production	In vivo study	[277]
IL‐2	T‐cell activation	Enhance IFN‐γ production but may also expand Treg cells	Clinical evaluation	[278]
Immune checkpoint inhibitors	PD‐1/CTLA‐4/TIM‐3	Partially restores multifunctional T‐cell responses	In vivo study	[279]
Luteolin	T‐cell differentiation/memory formation	Promotes effector‐to‐central memory transition and strengthens Th1/Th17 responses	In vivo study	[280]

Abbreviations: AICAR, 5‐aminoimidazole‐4‐carboxamide ribonucleotide; AhpC/AhpD, alkyl hydroperoxide reductase C/D; AMPK, AMP‐activated protein kinase; Bcl‐2, B‐cell lymphoma 2; CD, cluster of differentiation; COX‐2, cyclooxygenase‐2; CTLA‐4, cytotoxic T‐lymphocyte‐associated protein 4; GM‐CSF, granulocyte‐macrophage colony‐stimulating factor; IDO, indoleamine 2,3‐dioxygenase; IFN‐γ, interferon‐γ; IL, interleukin; ManLAM, mannose‐capped lipoarabinomannan; MDSC, myeloid‐derived suppressor cell; MHC‐II, major histocompatibility complex Class II; miR‐1281, microRNA‐1281; mTORC1, mechanistic target of rapamycin complex 1; PD‐1, programmed cell death protein 1; PDE, phosphodiesterase; PknG, protein kinase G; PtpA, protein tyrosine phosphatase A; RNS, reactive nitrogen species; ROS, reactive oxygen species; SapM, secreted acid phosphatase M; SODs, superoxide dismutases; TFEB, transcription factor EB; Th, T helper; TIM‐3, T‐cell immunoglobulin and mucin‐domain containing‐3; Treg, regulatory T cell; TrxB2, thioredoxin reductase B2; VPS33B, vacuolar protein sorting‐associated protein 33B.

#### 4.2.1 Promoting Phagosome Maturation or Inducing Autophagy

Inhibition of phagosome maturation and suppression of autophagy are major mechanisms by which *M. tuberculosi*s maintains long‐term intracellular survival within macrophages. Accordingly, restoring these lysosome‐dependent degradative pathways has become an important focus of adjunctive therapies. In the context of phagosome maturation arrest, several *M. tuberculosis* virulence factors, including PknG, PtpA, and SapM, play key roles. PknG inhibits phagosome–lysosome fusion, and tetrahydrobenzothiophene derivatives have been shown to inhibit its activity, promote lysosomal trafficking of mycobacteria‐containing phagosomes, and enhance macrophage killing of intracellular bacilli [244]. PtpA also interferes with phagosome maturation by disrupting VPS33B‐mediated endolysosomal trafficking in host cells [245]. Recent evidence further indicates that PtpA can suppress GPX4 expression and induce ferroptosis in host cells, thereby facilitating bacterial virulence and dissemination [281]. Consistent with this findings, chalcone derivatives, such as (2E)‐1‐(2′‐hydroxyphenyl)‐3‐(1‐naphthyl)‐2‐propen‐1‐one, have been reported to inhibit PtpA activity and restrict intracellular bacterial replication [282]. SapM impairs phagosome maturation through hydrolysis of PI3P on the phagosomal membrane and preventing its accumulation. Several compounds, including L‐ascorbic acid, 2‐phospho‐L‐ascorbic acid, and trihydroxyphenyl‐containing molecules such as tyrphostin 51, galloflavin, and YM‐26734, have been shown to reduce SapM enzymatic activity [246, 247]. In addition, the ManLAM inhibitor ZXL1 alleviates ManLAM‐mediated suppression of DC maturation and increases the expression of molecules such as MHC‐II and CD40, thereby enhancing host immune clearance [248].

Beyond phagosome maturation, autophagy represents another major lysosome‐dependent degradative pathway and an important target for restoring the intracellular antimicrobial activity in host cells. Classical autophagy inducers, such as rapamycin and its derivative everolimus, promote autophagosome formation by inhibiting mTORC1, thereby enhancing macrophage bactericidal activity [249, 250]. By contrast, metformin and 5‐aminoimidazole‐4‐carboxamide ribonucleotide indirectly suppress mTORC1 through AMPK activation, leading to increased expression of autophagy‐related genes and enhanced clearance of *M. tuberculosis* [251, 252]. Lipid‐lowering agents such as statins, including simvastatin, can also induce autophagy through activation of the AMPK–mTORC1–TFEB axis, thereby promoting intracellular bacterial clearance [253]. In addition, 1,25‐(OH)_2_D_3_ stimulates the expression of the antimicrobial peptide cathelicidin through the vitamin D receptor and further activates autophagy‐related molecules such as beclin‐1 and Atg5 [254]. Notably, several small molecules derived from traditional Chinese medicine (TCM), including baicalin, epigallocatechin‐3‐gallate, and honokiol, have also demonstrated the capacity to activate autophagic pathways [255, 256, 257]. Taken together, therapeutic strategies targeting phagosome maturation and autophagy provide a direct means of restoring macrophage intracellular clearance mechanisms in tuberculosis, although further preclinical and clinical validation is needed before their therapeutic potential can be fully defined.

#### 4.2.2 Targeting Antioxidant Defenses

To resist host‐derived ROS and RNS, *M. tuberculosis* has evolved a robust antioxidant defense network, including superoxide dismutases (SODs), alkyl hydroperoxide reductase, the Trx system, and heme‐containing proteins involved in NO detoxification. Accordingly, therapeutic strategies aimed at disrupting bacterial antioxidant defenses or enhancing host oxidative antimicrobial activity have attracted increasing interest as therapeutic approaches.

Within the bacterial antioxidant machinery, SODs and alkyl hydroperoxide reductase represent particularly important targets. *M. tuberculosis* encodes two metal‐dependent SODs, SodA and SodC, which detoxify superoxide radicals and contribute to bacterial survival under macrophage‐derived oxidative stress [38]. Pharmacological inhibition of SOD activity may therefore weaken the capacity of *M. tuberculosis* to withstand host‐derived ROS. In this context, dithiocarbamate compounds such as diethyldithiocarbamate and pyrrolidine dithiocarbamate have shown antimycobacterial activity against both replicating and nonreplicating *M. tuberculosis* [258]. Although these compounds are not established as specific inhibitors of SodA or SodC, their metal‐chelating properties may interfere with metal‐dependent antioxidant defenses and thereby sensitize bacilli to oxidative stress. The alkyl hydroperoxide reductase system, particularly AhpC and its associated reductase component AhpD, protects bacilli against organic hydroperoxides and peroxynitrite and may partially compensate for defects in other antioxidant pathways. Studies have shown that testosterone 3(E),17‐dioxime can inhibit AhpD to block electron transfer to AhpC, thereby weakening the ability of *M. tuberculosis* to eliminate lipid peroxides and making it more susceptible to host ROS attack [259]. These findings support the feasibility of targeting AhpC/AhpD‐dependent peroxide defense, although this strategy remains at an early experimental stage.

The Trx system represents another promising redox vulnerability. Trx reductase TrxB2 is essential for thiol‐redox homeostasis in *M. tuberculosis*, and genetic depletion of TrxB2 impairs bacterial fitness during infection. Several Trx‐system inhibitors or repurposed redox‐active compounds, including auranofin [260], ebselen [261], and PX‐12 [262], have been explored as antimicrobial candidates, although their specificity and therapeutic window in tuberculosis require further validation. In addition, AC2P36 [263], a thiol‐depleting compound, selectively kills *M. tuberculosis* under acidic conditions and potentiates the bactericidal activity of isoniazid, clofazimine, and diamide. These findings support the concept that pharmacological interference with bacterial thiol metabolism may sensitize bacilli to host antimicrobial stress and enhance the activity of existing antituberculosis drugs.

Another complementary approach is to enhance oxidative pressure within infected host cells. Several redox‐active agents have been reported to promote oxidative damage in *M. tuberculosis*. Vitamin C, for instance, can drive Fenton chemistry and sterilize drug‐susceptible and drug‐resistant *M. tuberculosis* cultures in vitro, and it can potentiate the bactericidal activity of first‐line antituberculosis drugs [264]. Clofazimine also exerts antimycobacterial effects, at least in part, through redox cycling and the generation of ROS [265]. In addition, IFN‐γ and GM‐CSF enhances ROS/RNS release by promoting macrophage maturation and activation and has shown potential to improve the efficacy of antituberculosis drugs in animal models [266, 267, 268].

These findings suggest that strategies designed to either inhibit bacterial antioxidant defenses or amplify pro‐oxidant antimicrobial stress may improve the clearance of persistent bacilli. However, because excessive oxidative stress can also aggravate host tissue injury and contribute to lung pathology, future studies should carefully define the optimal timing, dosage, and cellular context of redox‐based interventions.

#### 4.2.3 Remodeling Host Cell Death Programs: Promoting Protective Cell Death While Limiting Inflammatory Damage

*M. tuberculosis* can promote its survival and dissemination by reprogramming host cell death pathways. In general, virulent *M. tuberculosis* strains tend to suppress apoptosis, a relatively host‐protective form of cell death that facilitates pathogen clearance, while promoting necrotic and highly inflammatory forms of cell death that favor bacterial release and spread. Accordingly, therapeutic modulation of host cell death programs has emerged as an important direction in host‐directed therapies (HDTs). In the context of restoring protective apoptosis, *M. tuberculosis* can upregulate host Bcl‐2 family proteins to inhibit apoptotic signaling, whereas the small‐molecule Bcl‐2 inhibitor navitoclax, when combined with standard antituberculosis drugs, has been shown to enhances bacterial clearance in a mouse model [269]. Conversely, cyclophilin D (CypD)‐mediated mitochondrial permeability transition contributes to *M. tuberculosis*‐induced necrotic cell death. Inhibition of CypD with cyclosporin A can alleviate macrophage injury. In addition, miR‐1281 has been reported to exert a protective effect by negatively regulating CypD expression [270].

#### 4.2.4 Targeting the MDSC‐Mediated Immunosuppressive Microenvironment

Persistent survival of *M. tuberculosis* is facilitated in part by remodeling of the host immune microenvironment, in which MDSCs serving as key mediators of immunosuppression. Through high‐level expression of ARG1 and iNOS, MDSCs deplete local arginine and generate ROS and NO, thereby disrupting TCR signaling and suppressing T‐cell activation and proliferation, ultimately driving T‐cell dysfunction [283, 284]. MDSCs can also secrete IL‐10 and TGF‐β, which inhibit IFN‐γ production by Th1 cells and further reinforce the immunosuppressive milieu. Consequently, therapeutic targeting of MDSCs has emerged as an important strategy for reversing immune suppression in tuberculosis.

Several approaches have been proposed to alleviate MDSC‐mediated immunosuppression. Arginase1 (ARG1) inhibitors can restore arginine metabolic balance and strengthen antituberculosis immunity, while agents targeting IDO, such as 1‐methyl‐D‐tryptophan and COX‐2 inhibitors, may likewise attenuate MDSC‐associated suppression [271, 272]. In addition, phosphodiesterase inhibitors have shown potential for modulating MDSC function; notably, roflumilast has been reported to enhance the bactericidal efficacy of isoniazid [273]. Metabolic targeting represents another promising approach: JHU083, a glutamine antagonist, can both restrict host‐derived glutamine availability to *M. tuberculosis* and reduces suppressive myeloid populations while increasing effector T cells, leading to reduced lung bacterial burden and improved pulmonary pathology [274]. Taken together, targeting MDSCs represents a promising HDT strategy for tuberculosis by reshaping the immunosuppressive microenvironment and restoring protective antimycobacterial immunity.

#### 4.2.5 Enhancing T‐Cell Immune Responses

T‐cell dysfunction is a defining immunological feature of chronic tuberculosis, making restoration of T‐cell quantity and effector function an important objective of HDTs. Among the available approaches, cytokine‐based therapy represents one of the most direct strategies for enhancing protective cellular immunity. IL‐7 has been shown to promote CD4^+^ T‐cell proliferation and expand the naïve and central memory T‐cell compartments, suggesting its potential utility in settings characterized by T‐cell impairment, such as HIV‐associated tuberculosis [275]. IL‐15, particularly when combined with IL‐7 or BCG, can augment CD4^+^ and CD8^+^ T‐cell responses, reduce pulmonary bacterial burden, and reinforce Th1 immunity [276]. IL‐12 also plays a critical role in antimycobacterial defense by promoting Th1 differentiation and stimulating IFN‐γ production, thereby contributing to granuloma formation and host protection [277]. By contrast, although IL‐2 can increase IFN‐γ production, it may also promote Treg expansion, highlighting the need for careful evaluation of its therapeutic application [278]. Overall, cytokine‐based therapy holds promise for restoring T‐cell immunity in tuberculosis; however, its therapeutic benefit will depend on achieving an appropriate balance between protective immune activation and immunopathological responses.

Beyond cytokine supplementation, reversal of T‐cell exhaustion has emerged as another important direction in antituberculosis immunotherapy. During chronic *M. tuberculosis* infection, persistent antigen exposure drives sustained expression of inhibitory receptors, including PD‐1, CTLA‐4, and TIM‐3, on antigen‐specific T cells, leading to reduced proliferative capacity and impaired effector function [285]. In vitro studies have shown that blockade of these immune checkpoints can partially restore multifunctional T‐cell responses, with PD‐1 blockade producing the most pronounced effect [279]. However, in a rhesus macaque model, PD‐1 blockade enhanced CD8^+^ T‐cell activity but was also associated with increased bacterial burden in granulomas and exacerbated inflammatory pathology [286]. These findings showed that checkpoint‐targeted intervention in tuberculosis requires considerable caution.

In addition to biologics, certain small‐molecule compounds derived from TCM have also shown potential for enhancing T‐cell immunity. For example, luteolin has been reported to promote the transition from effector memory T cells to central memory T cells and to strengthen Th1 and Th17 responses [280]. thereby improving host clearance of *M. tuberculosis* and potentially shortening the duration of standard antituberculosis therapy [280]. These effects suggest that TCM may serve as a promising HDT candidate for enhancing host clearance of *M. tuberculosis* and may have the potential to shorten the duration of standard antituberculosis therapy.

## 5 Conclusion and Prospects: Knowledge Gaps and Emerging Technologies for Future Research

### 5.1 Spatial Immunology of Granulomas

Tuberculous granulomas are initially organized as host‐protective structures that restrict the dissemination of *M. tuberculosis*. However, *M. tuberculosis* can also exploit their unique microenvironment, including caseous necrosis, hypoxia, lipid accumulation, and fibrotic remodeling, to create an “immune shelter” niche that supports long‐term persistence. Previous studies have shown that *M. tuberculosis* employs stage‐specific immune evasion strategies during infection. For instance, during early infection, the bacillus interferes with phagosome maturation and delays T‐cell priming, whereas at later stages it contributes to the establishment of an immunosuppressive and metabolically constrained granulomatous microenvironment. However, most of these mechanisms have been investigated predominantly in single‐cell‐type models or simplified experimental systems. Consequently, the precise lesion compartments, cellular neighborhoods, and temporal windows in which these immune evasion processes occur remain incompletely defined and insufficiently integrated.

Future studies should move beyond traditional immunological analyses on individual cell types and instead investigate the lesion microenvironment. A major priority will be to define how the multiple immune evasion strategies orchestrated by *M. tuberculosis* shape the formation, maintenance, and dynamic evolution of tuberculous lesions, thereby providing a conceptual foundation for the development of HDTs. The integration of multilayered spatial profiling technologies will be critical in addressing these questions. First, combined single‐cell and spatial transcriptomic analyses can directly link cellular states with their anatomical localization within lesions. This approach is particularly valuable for determining how immune cell populations distincted across distinct granuloma compartments contribute either to bacterial containment or to tissue damage. Second, integration of imaging mass cytometry with multiomics approaches will facilitate the construction of an immunometabolic atlas of tuberculosis granulomas, providing insights into how oxygen tension, lipid metabolism, cytokine gradients, and distinct cellular niches collectively shape lesion fate.

### 5.2 Systematic Investigation of Combinatorial Immune Correlates of Vaccine Protection

One of the greatest bottlenecks in the development of new tuberculosis vaccines is the lack of validated CoPs [287]. Historically, tuberculosis vaccine research has relied mainly on the assumption that Th1/IFN‐γ responses could serve as surrogate markers of protective immunity. Although IFN‐γ is indispensable for host defense against mycobacterial infection, enhancement of Th1 responses alone does not reliably predict vaccine efficacy. MVA85A is a representative example: Despite inducing strong Th1 immunogenicity in both animal models and humans, it failed to confer significant protection in a Phase IIb clinical trial [229].

The future development of tuberculosis vaccine will depend critically on the identification of immunological indicators that can reliably predict protective efficacy. Such correlates are unlikely to be captured by a single IFN‐γ measurement or isolated T‐cell readout, but rather are expected to emerge from the integrated interplay of innate immunity, adaptive immunity, and local pulmonary immune responses. Given the central role of mycobacterial cell wall glycolipids in immune evasion, and the fact that many of these antigens are presented through noncanonical antigen‐presentation pathways, future vaccine design and evaluation should place greater emphasis on these antigenic targets, as well as on the trained immunity and humoral responses they elicit. Moreover, with the rapid development of multiomics technologies and artificial intelligence‐driven analytics, integration of multimodal data generated from robust tuberculosis granuloma models is becoming increasingly feasible, creating new opportunities for the identification of composite biomarkers.

### 5.3 Models for the Study of Tuberculosis Granulomas

The lacking of animal models that faithfully recapitulate liquefactive necrosis and cavitation in human tuberculous granulomas has substantially hampered investigations into tuberculosis‐associated immunopathological mechanisms and the development of effective intervention strategies. To address this limitation, our group previously established a rabbit skin model of tuberculous granuloma using multiple mycobacterial strains. Notably, rabbit skin exhibited high susceptibility to *M. bovis*, and even intradermal inoculation with BCG strain was sufficient to induce granulomatous lesions characterized by liquefactive necrosis and cavitary changes [288]. Using this model, we subsequently evaluated the antituberculosis efficacy of several immunotherapeutic agents and vaccine candidates [289, 290], highlighting its potential as a useful platform for studying granuloma formation, lesion progression, and therapeutic intervention in tuberculosis.

Meanwhile, recently developed lung organoids infection models have begun to partially recapitulate the early events of *M. tuberculosis* infection in pulmonary epithelial cells and macrophages, and have been used for the preliminary evaluation of drug candidates [291]. Furthermore, respiratory organ‐on‐a‐chip platforms, by integrating key physiological features such as mechanical stimulation, vascular interfaces, and immune cell migration, provide experimental systems that more closely approximate the human pulmonary conditions [292]. These platforms offer valuable opportunities to investigate granuloma formation, remodeling of the local immune microenvironment, and drug responses under more physiologically relevant conditions. Accordingly, the combined application of complementary research platforms that better mimic the pathological processes of human tuberculosis, including the rabbit skin liquefactive granuloma model, organoid systems, and microfluidic chip models, will provide important support for elucidating tuberculosis immunopathogenesis and developing novel intervention strategies.

### 5.4 TCM in HDTs for Tuberculosis

HDTs has emerged as an important adjunct to antimicrobial chemotherapy for tuberculosis, yet its clinical translation remains challenging. *M. tuberculosis* subverts host immune defense through multiple bacterial components and molecular pathways, resulting in impaired phagocytic killing, the establishment of an immunosuppressive microenvironment, and attenuation of protective T‐cell responses. Accordingly, effective antituberculosis HDT is unlikely to be achieved through a single‐target approach alone. Instead, it may require combinatorial strategies that enhance intracellular bacterial clearance, remodel the immunosuppressive microenvironment, and reinforce adaptive immunity. However, the combined use of multiple chemical agents may increase the risk of toxicity and adverse effects, thereby limiting broader clinical application.

In this context, TCM may offer distinctive potential in HDTs for tuberculosis, because of its multicomponent, multitarget, and system‐level regulatory properties. First, TCM can simultaneously modulate multiple layers of host defense, making it potentially well suited to the complex and multilayered host–pathogen interaction network underlying tuberculosis pathogenesis. Second, certain TCM‐derived interventions may have advantages in tolerability during prolonged treatment, although they still requires rigorous validation in well‐designed preclinical and clinical studies. Accumulating evidence indicates that several bioactive compounds derived from Chinese medicinal herbs, such as baicalin and luteolin [255, 280, 293], can enhance host control of *M. tuberculosis* and shorten the standard antituberculosis therapy. Collectively, these findings suggest that TCM may represent an important complementary approach to antituberculosis therapies.

## Author Contributions

XJZ: writing – original draft, literature review, and visualization. YL: literature review and visualization. YLH: literature review. HXN: conceptualization, writing – original draft, and writing – review and editing. All authors have read and approved the final manuscript.

## Ethics Statement

The authors have nothing to report.

## Conflicts of Interest

The authors declare no conflicts of interest.

## Data Availability

The authors have nothing to report.
